# An Adaptable Framework for Factors Contributing to Medication Adherence: Results from a Systematic Review of 102 Conceptual Frameworks

**DOI:** 10.1007/s11606-021-06648-1

**Published:** 2021-03-03

**Authors:** Kai Qi Elizabeth Peh, Yu Heng Kwan, Hendra Goh, Hasna Ramchandani, Jie Kie Phang, Zhui Ying Lim, Dionne Hui Fang Loh, Truls Østbye, Dan V. Blalock, Sungwon Yoon, Hayden Barry Bosworth, Lian Leng Low, Julian Thumboo

**Affiliations:** 1 Duke-NUS Medical School, Singapore, Singapore; 2 Program in Health Services and Systems Research, Duke-NUS Medical School, Singapore, Singapore; 3 Department of Pharmacy, Faculty of Science, National University of Singapore, Singapore, Singapore; 4 Department of Rheumatology & Immunology, Singapore General Hospital, Singapore, Singapore; 5 SingHealth Regional Health System, Singapore Health Services, Singapore, Singapore; 6 Department of Family Medicine and Community Health, Duke University, Durham, NC USA; 7 Department of Psychiatry and Behavioral Sciences, Duke University School of Medicine, Durham, NC USA; 8 Center of Innovation to Accelerate Discovery and Practice Transformation (ADAPT), Durham Veterans Affairs Health Care System, Durham, NC USA; 9 Departments of Population Health Sciences and Psychiatry and Behavioral Sciences, School of Medicine, School of Nursing, Duke University, Durham, NC USA; 10 SingHealth Duke-NUS Family Medicine Academic Clinical Program, Singapore, Singapore; 11 Department of Family Medicine and Continuing Care, Singapore General Hospital, Singapore, Singapore; 12 PULSES Centre Grant, SingHealth Regional Health System, Singapore, Singapore; 13 Post-Acute and Continuing Care, Outram Community Hospital, Singapore, Singapore; 14 Department of Biology, Faculty of Science, National University of Singapore, Singapore, Singapore

## Abstract

**Objective:**

To summarize the available conceptual models for factors contributing to medication adherence based on the World Health Organization (WHO)’s five dimensions of medication adherence via a systematic review, identify the patient groups described in available conceptual models, and present an adaptable conceptual model that describes the factors contributing to medication adherence in the identified patient groups.

**Methods:**

We searched PubMed®, Embase®, CINAHL®, and PsycINFO® for English language articles published from inception until 31 March 2020. Full-text original publications in English that presented theoretical or conceptual models for factors contributing to medication adherence were included. Studies that presented statistical models were excluded. Two authors independently extracted the data.

**Results:**

We identified 102 conceptual models, and classified the factors contributing to medication adherence using the WHO’s five dimensions of medication adherence, namely patient-related, medication-related, condition-related, healthcare system/healthcare provider-related, and socioeconomic factors. Eight patient groups were identified based on age and disease condition. The most universally addressed factors were patient-related factors. Medication-related, condition-related, healthcare system-related, and socioeconomic factors were represented to various extents depending on the patient group. By systematically examining how the WHO’s five dimensions of medication adherence were applied differently across the eight different patient groups, we present a conceptual model that can be adapted to summarize the common factors contributing to medication adherence in different patient groups.

**Conclusion:**

Our conceptual models can be utilized as a guide for clinicians and researchers in identifying the facilitators and barriers to medication adherence and developing future interventions to improve medication adherence.

**Protocol Registration:**

PROSPERO Identifier: CRD42020181316

**Supplementary Information:**

The online version contains supplementary material available at 10.1007/s11606-021-06648-1.

## INTRODUCTION

Medication adherence is defined as the process by which patients take their medications as prescribed, described by three phases: initiation, implementation, and discontinuation.^1^ Suboptimal adherence is a very common phenomenon. Average adherence to medication ranges from 50 to 79% among patients suffering from chronic diseases.^2–4^ Appropriate and optimal prescription drug use is a major public health challenge. Poor adherence can compromise the effectiveness of treatment, making adherence a problem of increasing concern in terms of health outcomes and healthcare costs.^2^ Overutilization and underutilization of medications are arguably equally important, at least in high-income countries. This article addresses underutilization.

Medication adherence is a complex behavior influenced by patient-related factors, the healthcare team/system, characteristics of the disease, treatment, and social and economic factors.^2^ It has been observed that adherence is typically higher among patients with acute conditions, as compared to those with chronic conditions.^5, 6^ A greater degree of adherence to medication is associated with effective therapeutic regimens for cure, as compared to treatments aimed at prevention.^7, 8^ However, when medication is to be taken over a long period, adherence drops substantially for both prevention and cure.^8^ Adherence to medications also varies by age; younger patients appear to have better adherence than older patients.^9^ In children, adherence to drug therapy is also affected by their dependence on an adult caregiver.^8^

The factors contributing to medication adherence have been widely studied. Many conceptual models have been developed to help understand the factors contributing to medication adherence in specific patient groups and/or for specific disease conditions.^10–12^ A number of theoretical approaches^13, 14^ including the Health Belief Model, Social Cognitive Theory, Theory of Reasoned Action, Theory of Planned Behavior, and the Trans-Theoretical Model have also been employed. While these theories are helpful in understanding the contribution of patient-related factors and community or environment in medication adherence, they often ignore the effect of healthcare system– and healthcare team–related factors on patient behavior towards medication adherence.^11^ Clinicians and researchers may also find it difficult to implement the published frameworks in their own clinical practice as the clinical context and patient group may differ across studies.

Therefore, we aimed to (1) summarize the available conceptual models for factors contributing to medication adherence based on WHO’s five dimensions of medication adherence via this systematic review, (2) identify the patient groups described in available conceptual models, and (3) present an adaptable model that describes the factors contributing to medication adherence in the identified patient groups. The goal is for our conceptual models to assist clinicians and researchers to better understand and improve medication adherence in the patient group of interest.

## METHODS

This systematic review was guided by the preferred reporting items for systematic review and meta-analysis (PRISMA) statement.^15^

### Search Strategy

We searched PubMed®, Embase®, CINAHL®, and PsycINFO® for English-language papers published until 31 March 2020. A medical librarian was consulted for the design of the search strategy. The search strategy used keywords relevant to medication adherence and a theoretical or conceptual framework. The specific search strategy can be found in Supplementary Tables 1–4.

### Article Selection

All titles and abstracts were screened independently by two reviewers (KQEP, HR). A third reviewer (YHK) was consulted when a disagreement arose between the two reviewers. For articles that were potentially relevant, the full text of these articles was independently reviewed by two reviewers (KQEP, HG) for inclusion or exclusion. We included articles if they were full-text original publications in English and presented theoretical or conceptual models for factors contributing to medication adherence. We excluded articles that presented statistical models without a clear conceptual foundation. We also excluded unpublished articles, conference abstracts, expert opinions, or book chapters. Animal studies, case studies, and non-English studies were also excluded.

### Data Extraction

Where available, the following data elements were independently extracted by two reviewers (KQEP, HG): (1) objective; (2) characteristics of the study population: country of study, sample size, age, gender, disease condition; (3) factors related to medication adherence; (4) whether the model used was based on literature, empirical data, or another source; (5) salient themes; (6) gaps of the model.

### Synthesis of Results

We classified the factors contributing to medication adherence in each model using the WHO’s five dimensions of medication adherence^2^ as it is a widely used framework that provides a holistic approach to understanding medication adherence.^16–19^ The five dimensions are patient-related factors, medication-related factors, condition-related factors, healthcare system/healthcare provider (HCP)-related factors, and socioeconomic factors. We identified prominent patient groups in our included studies based on age (adult/pediatrics) and type of disease condition as medication adherence varies by age,^9^ and the type of disease condition may influence perceived disease threat and health risk,^20^ which in turn impacts adherence behavior.^21^ The review team discussed and synthesized information in an iterative process, considering the strengths and weaknesses of each conceptual model, as well as common factors and gaps across models in each patient group. Finally, we present a “donut model” that illustrates the common factors contributing to medication adherence based on WHO’s five dimensions of medication adherence and applied our model to the patient groups identified to describe the factors contributing to medication adherence specific to the identified patient groups.

## RESULTS

**Figure 1:**
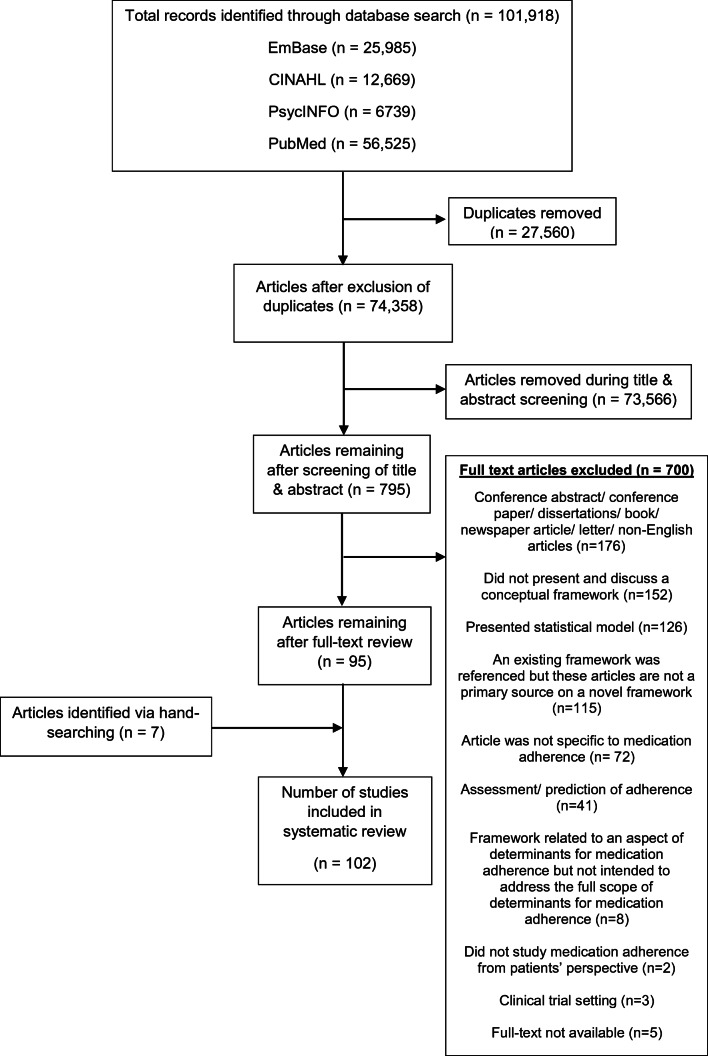
PRISMA flow diagram for systematic review.

A search on PubMed®, Embase®, CINAHL®, and PsycINFO® for English-language papers published until 31 March 2020 yielded a total of 101,918 studies, of which 27,560 duplicates were excluded. A review of the titles and abstracts further excluded 73,566 studies as they did not meet the inclusion criteria. The remaining 795 papers underwent full-text review by two reviewers, and 700 articles were further excluded, with reasons provided in Figure 1. Hand-searching of reference lists yielded 7 additional studies, resulting in 102 relevant studies for final inclusion in this systematic review.

### Purpose of Existing Models

The 102 studies presented unique conceptual models for factors contributing to medication adherence for the following purposes: (i) gain a deeper understanding of factors contributing to medication adherence (*n* = 64); (ii) inform interventions, instrument development, or future adherence studies (*n* = 30); (iii) describe the decision making process behind medication adherence (*n* = 4); (iv) allow better visualization of broad categories of factors contributing to medication adherence (*n* = 3); and (v) identify patients at risk of nonadherence (*n* = 1).

### Common Factors and Gaps Across Models by Patient Groups

We identified eight patient groups described in available conceptual models: (1) adults with chronic, non-communicable conditions, e.g., hypertension, hyperlipidemia, diabetes mellitus; (2) adults with cancer; (3) adults with chronic, non-communicable conditions with asymptomatic and flare phases, e.g., rheumatoid arthritis and asthma; (4) adults with symptomatic conditions such as nocturia and migraine; (5) adults undergoing treatment for chronic, communicable conditions, e.g., human immunodeficiency virus (HIV) and tuberculosis; (6) adults taking medication for prevention of communicable diseases, e.g., HIV and tuberculosis; (7) adults with psychiatric conditions, e.g., schizophrenia; and (8) pediatrics patients. The common factors contributing to medication adherence in these patient groups are summarized in Table 1. A summary of the 102 conceptual frameworks can be found in Supplementary Tables 5–14.

**Table 1:** Common Factors Contributing to Medication Adherence Across Models in Various Patient Groups

Patient group	Patient/caregiver-related factors	Condition-related factors	Medication-related factors	Healthcare system/HCP-related factors	Socioeconomic factors
Conceptual frameworks not specific to any patient group (*n* = 11)^22–32^	*n* = 11^22–32^	*n* = 4^24, 29, 31, 32^	*n* = 7^23–26, 30–32^	*n* = 8^22, 24–26, 29–32^	*n* = 7^22, 25, 28–32^
	Cognitive and psychological factors (i.e., perceptions, beliefs, concerns, knowledge/health literacy, emotions, evaluation of medication, prospect theory, forgetful, loss of interest, conserve supply and reduce cost, commitment, motivation, acceptance, patient preference, health decision) (*n* = 9); Patient characteristics (e.g., demographics, experience, type of user**)** (*n* = 3); Behavioral factors (i.e., organization, planning, lifestyle, interaction with HCP/healthcare system) (*n* = 2);	Symptoms (*n* = 3); Health outcome (*n* = 1); Experience with disease (*n* = 1)	Medication effects (i.e., experience, benefits, drawbacks) (*n* = 4); Medication regimen (i.e., unclear label instructions, regimen familiarity, complexity, dosage, characteristics) (*n* = 3); Medication cost (*n* = 2)	HCP characteristics (i.e., relationship, communication, ability to relate, provision of information, bilateral bargaining theory, interaction with HCP/healthcare system) (*n* = 8)	Social/environmental factors (i.e., social context, interaction, support, culture, language, peer group norms, external influences, sociodemographic, promotional prompts, practical problems) (*n* = 6); Economic factors (i.e., consumer choice theory) (*n* = 1)
Adults with chronic, non-communicable conditions, e.g., hypertension, hyperlipidemia, diabetes mellitus (*n* = 30)^10–12, 33–59^	*n* = 30^10–12, 33–59^	*n* = 15^10–12, 34, 35, 41, 42, 45, 49, 51–53, 55, 56, 58^	*n* = 17^11, 12, 33, 34, 36, 38, 40–42, 45, 49, 52–56, 59^	*n* = 22^10–12, 33, 36–38, 40–49, 51, 52, 54, 56, 59^	*n* = 21^10–12, 33–35, 40–46, 48–52, 54, 56, 59^
	Cognitive and psychological factors (i.e., perception, beliefs, concerns, knowledge, emotions, intentional and unintentional non-adherence, expectation of treatment, motivation, skills, faith, risk estimation) (*n* = 27); Patient characteristics (e.g., demographics, physical function, experience, type of user, treatment responsibility) (*n* = 17); Behavioral factors (i.e., behavioral characteristics, organization, self-regulatory fatigue, integration of medication routine into lifestyle) (*n* = 9); Priorities (i.e., competing needs, QoL) (*n* = 5)	Disease control (i.e., symptoms, complications, severity, acute events) (*n* = 9); Patient-specific (i.e., co-morbidities, family history, past medical history, medical/disability-related) (*n* = 6); Other disease characteristics (i.e., context, duration, type, effect on QoL, effect on life expectancy) (*n* = 4)	Medication effects (i.e., side effects, effectiveness, risks and benefits) (*n* = 12); Medication regimen (i.e., complexity, dosing, type, pill burden, drug class, frequency, interference in routine) (*n* = 12); Patient-specific issues (i.e., experience, acceptability, time between diagnosis and treatment, past medication history, logistics issue, injection site issue, needle phobia) (*n* = 7); Other medication properties (cost, tablet supply, packaging) (*n* = 5)	HCP factors (i.e., relationship, interaction, quality of care, patient education, shared decision making) (*n* = 19); Healthcare system characteristics (i.e., accessibility, policies, affordability, provider continuity) (*n* = 14)	Social/environmental factors (i.e., social support, stigma, culture, access, vicarious experience, interpersonal influence, life status changes) (*n* = 16); Economic factors (i.e., financial constraints, socioeconomic status) (*n* = 9); Lifestyle factors (i.e., alcohol/drug use) (*n* = 2)
Adults with cancer (*n* = 7)^60–66^	*n* = 7^60–66^	*n* = 4^60–62, 66^	*n* = 6^60, 62–66^	*n* = 7^60–66^	*n* = 6^61–66^
	Cognitive and psychological factors (i.e., perception, belief, concerns, knowledge, self-efficacy, expectation of pain relief, denial of pain as symptom, decision making process, goals and values, emotions, psychological factors, unintentional adherence) (*n* = 7); Patient and family characteristics (i.e., demographics, physical factors, family hesitancy, family characteristics) (*n* = 4); Behavioral factors (i.e., skills, management) (*n* = 2); Priorities (i.e., balancing quantity of life and quality of life) (*n* = 2)	Disease control (i.e., feeling better, illness recurrence/metastasis, impact on quality of life) (*n* = 3); Disease characteristics (i.e., time since diagnosis, risk of pregnancy, complexity, complications) (*n* = 1); Co-morbidities (*n* = 1)	Medication effects (i.e., side effects, efficacy, treatment outcome, satisfaction, impact on lifestyle/emotions) (*n* = 6); Medication regimen (i.e., type of analgesic, complexity, dose, duration, drug class, concomitant medications) (*n* = 3); Medication properties (i.e., physical properties, cost) (*n* = 2)	HCP characteristics (i.e., relationship, communication, clinical care, duration of visit, prescribing practice, race disparity, selection of appropriate patients for oral therapy) (*n* = 7); Healthcare system factors (i.e., obtaining analgesics, regulation processes, insurance, prescription coverage, reimbursement, fragmented system, regular information support) (*n* = 3)	Social/environment factors (i.e., social support, socio-cultural factors, environment) (*n* = 5); Lifestyle factors (i.e., social situations) (*n* = 1)
Adults with chronic, non-communicable conditions with asymptomatic and flare phases, e.g., rheumatoid arthritis and asthma (*n* = 6)^67–72^	*n* = 6^67–72^	*n* = 3^68, 70, 71^	*n* = 4^68–71^	*n* = 4^67–70^	*n* = 3^69, 70, 72^
	Cognitive and psychological factors (i.e., perceptions, beliefs, concerns, knowledge, emotions, decision making process, motivation, goals, skills, memory, attention, self-efficacy, expectation of outcome) (*n* = 6); Patient characteristics (i.e., experience, caregiver issues, demographics, personality) (*n* = 4)	Disease control (i.e., symptoms, acute events, sensation, impact on lifestyle) (*n* = 3); Patient-specific factors (i.e., mental health) (*n* = 1); Disease characteristics (i.e., prognosis) (*n* = 1)	Medication effects (i.e., side effects, effectiveness) (*n* = 3); Medication regimen (i.e., convenience, choice of drugs, interference in daily routine, treatment plan) (*n* = 3); Patient-specific factors (i.e., experience, acceptability) (*n* = 2); Other medication properties (i.e., change of name/appearance, cost) (*n* = 1)	HCP characteristics (i.e., relationship, care, communication, counselling) (*n* = 4); Healthcare system characteristics (i.e., issues, drug supply, access) (*n* = 2)	Social/environment factors (i.e., family/social support, culture, others’ views) (*n* = 3); Economic factors (cost, insurance) (*n* = 2)
Adults with symptomatic conditions, e.g., nocturia and migraine (*n* = 2)^73, 74^	*n* = 2^73, 74^	*n* = 2^73, 74^	*n* = 2^73, 74^	*n* = 2^73, 74^	*n* = 1^74^
	Cognitive factors (i.e., attitude, belief, knowledge, self-efficacy) (*n* = 2); Patient characteristics (i.e., age, sex, race, ethnicity) (*n* = 1)	Co-morbidities (*n* = 1); Symptoms bother (*n* = 1); Importance (*n* = 1)	Medication effects (i.e., side effects, efficacy, safety) (*n* = 2); Medication regimen (i.e., number, type, frequency, duration, follow-up care) (*n* = 1)	HCP factors (i.e., communication, trust) (*n* = 2); Healthcare system characteristics (i.e., continuity of care, wait time, volume) (*n* = 1)	Geographic/environmental factors (*n* = 1); Economic factors (i.e., income, insurance) (*n* = 1)
Adults undergoing treatment for chronic, communicable conditions, e.g., HIV, tuberculosis in resource-limited countries, e.g., Africa, Papua New Guinea (*n* = 9)^75–81^	*n* = 9^75–81^	*n* = 5^75, 77, 81–83^	*n* = 4^75, 77, 81, 82^	*n* = 7^75–77, 79, 80, 82, 83^	*n* = 9^75–81^
	Cognitive and psychological factors (i.e., beliefs, attitude, motivation, self-efficacy, information, expectation, acceptance, resilience, confidence, desire to be healthy, faith, emotions) (*n* = 9); Behavioral factors (i.e., skills, use of alternative treatment) (*n* = 6); Other personal characteristics (i.e., physical, social, and mental dimensions of health) (*n* = 1)	Patient-specific factors (i.e., long history of suffering, previous or current related illness) (*n* = 3); Disease control (i.e., symptoms, prevention of transmission to child, CD4 count) (*n* = 2);	Medication effects (i.e., side effects, consequences of non-adherence, effectiveness) (*n* = 4); Medication properties (i.e., scientific uncertainty, lifelong nature) (*n* = 2)	HCP characteristics (i.e., authoritarian HCP, clinic staff support, trust in provider, inconsistency in patient education, reinforcement) (*n* = 5); Healthcare system factors (i.e., quality of health services, medical system, governance) (*n* = 4)	Social/environmental factors (i.e., social relationship, social/community/institutional support, family and social responsibility, practical/structural barriers, social identity, gender norms, conflicting information, external support, stigma, discrimination, sociocultural policy) (*n* = 9); Economic factors (i.e., socioeconomic factors, poverty) (*n* = 3); Lifestyle factors (i.e., substance abuse) (*n* = 2)
Adults undergoing treatment for chronic, communicable conditions, e.g., HIV, tuberculosis from empirical data, existing theories and in countries, e.g., USA, Europe, Taiwan (*n* = 12)^84–95^	*n* = 12^84–95^	*n* = 6^85, 91–95^	*n* = 6^85, 89, 92–95^	*n* = 6^85, 89–92, 94^	*n* = 11^84–92, 94, 95^
	Cognitive and psychological factors (i.e., knowledge, motivation, perceptions, attitude, concerns, self-identity, values, conscious engagement, psychological health, emotions, acceptance, personal meaning of time and quality of life) (*n* = 11); Behavioral factors (i.e., skills, non-disclosure, lifestyle – demands and organization) (*n* = 8); Patient characteristics and experience (*n* = 2)	Patient specific (i.e., general health, co-morbidity, experience, illness representation) (*n* = 5); Disease control (i.e., signs, symptoms, fickle medical markers) (*n* = 4); Other characteristics (i.e., health outcome, silent virus, attributional uncertainty) (*n* = 3)	Medication effects (i.e., side effects, effectiveness, impact on lifestyle) (*n* = 5); Medication regimen (i.e., complexity, burden, instructions, convenience) (*n* = 5); Other medication properties (i.e., physical features, cost) (*n* = 3); Patient-specific factors (i.e., experience, concurrent treatment regimens) (*n* = 2)	HCP characteristics (i.e., relationship, communication, attitude) (*n* = 4); Healthcare system characteristics (health insurance, access, issues) (*n* = 3)	Social/environmental factors (i.e., social support, influence, stigma, access, culture, family, norms, interaction, communication) (*n* = 11); Economic factors (i.e., living condition, income, education, occupation, material and structural challenges) (*n* = 3); Lifestyle factors (i.e., substance abuse) (*n* = 2)
Adults taking medications for prevention of communicable conditions, e.g., prevention of HIV, tuberculosis (*n* = 2)^96, 97^	*n* = 2^96, 97^	*n* = 1^96^	*n* = 0	*n* = 1^97^	*n* = 2^96, 97^
	Cognitive and psychological factors (i.e., knowledge, perception, attitude, personal intention) (*n* = 2); Behavioral factors (i.e., behavioral skills) (*n* = 1)	Psychological ill health (*n* = 1)	Not explicitly included in models	Efficiency of services (*n* = 1); Trust in healthcare system (*n* = 1); Resources to access healthcare (*n* = 1)	Social/environmental factors (i.e., social support, pre-exposure prophylaxis skepticism (media/provider), social norms) (*n* = 2); Economic factors (i.e., insurance coverage, lack of stable housing) (*n* = 1); Lifestyle factors (i.e., substance use) (*n* = 1)
Adults with psychiatric conditions (*n* = 14)^98–111^	*n* = 14^98–111^	*n* = 10^100, 102–105, 107–111^	*n* = 13^98–101, 103–111^	*n* = 7^101, 102, 106–108, 110, 111^	*n* = 10^98–100, 103, 106–111^
	Cognitive and psychological factors (i.e., perceptions, beliefs, concerns, knowledge, functional ability, motivation, self-efficacy, values, assessment of options, forgetfulness, emotions, autonomy, acceptance, denial of disorder, psychological inflexibility, goals and priorities, idea of lifetime disorder, necessity of daily medications) (*n* = 14); Patient characteristics (i.e., personal experience, quality of life, health status, personal issues, loss of credible identity due to hospitalization) (*n* = 4); Behavioral factors (i.e., cues to act, participation in treatment) (*n* = 3)	Disease control (i.e. psychological distress, hospitalization, improvement in cognitive thinking, avoid psychoses) (n=6); Disease characteristics (i.e. cognitive deficit, impact on health, symptoms, lack of awareness, psychopathology) (n=6); Patient specific factors (i.e. medical conditions, reactance to disempowerment) (n=2)	Medication effects (i.e. side effects, effectiveness, avoid withdrawal symptoms, medication interaction, benefits, safety, experience) (n=13); Medication regimen (i.e. complexity, dose, inconvenience) (n=5); Medication cost (n=1)	HCP characteristics (i.e. relationship, collaborative decision making, insufficient information, attitude, ambivalence, reaction, patient education) (n=6); Healthcare system factors (i.e. escape from hospital, health system access, inadequacies) (n=4)	Social/ environmental factors (i.e. social support, practical barriers, stigma, culture, reaction from family, friends, caregiver availability) (n=10); Economic factors (i.e. lack of resources, living circumstances) (n=3); Lifestyle factors (i.e. substance abuse) (n=2)
Pediatrics patients (*n* = 9)^112–120^	*n* = 9^112–120^	*n* = 5^113, 114, 117, 118, 120^	*n* = 6^112, 113, 115, 117, 118, 120^	*n* = 5^113, 116, 118–120^	*n* = 8^112–116, 118–120^
	Personal characteristics (i.e., family, caregiver and child characteristics, child’s relationship with caregiver, adult support, child’s personal responsibility) (*n* = 6); Cognitive and psychological factors (i.e., beliefs, perception, information, emotion, caregiver acceptance, trust, autonomy, self-efficacy, motivation, plans for future, illness representation, appraisal) (*n* = 6); Behavioral factors (i.e., skills – administration routine, coping strategy, rewarding adherence, physical and psychological capability) (*n* = 5)	Disease control (i.e., number, severity of symptoms, Lazarus effect, prognosis, functional remission) (*n* = 3); Patient-specific factors (i.e., time since diagnosis, history of declining health, concrete thinking) (*n* = 3); Disease characteristics (i.e., identity, timeline, consequences) (*n* = 1)	Medication effects (i.e., side effects, effectiveness, experience) (*n* = 4); Medication properties (i.e., physical properties, formulation, cost) (*n* = 4); Medication regimen (i.e., dosing, duration, frequency, count, type, administration, complexity) (*n* = 3)	Healthcare system factors (i.e., access, delay, hospitalization, access to health insurance, drug supply adequacy, medical facility) (*n* = 5); HCP factors (i.e., relationship, skills, supportive presence, communication, treatment decision) (*n* = 3)	Social/environmental factors (i.e., social, community, institutional support, stigma, media portrayal, geographic variation, transportation, emotional and informational support, social and cultural characteristics, presence of more siblings) (*n* = 7); Economic factors (i.e., cost, socioeconomic status, caregiver’s economic resources, lack of resources) (*n* = 5)

*Abbreviations*: *HCP* healthcare provider, *HIV* human immunodeficiency virus, *QoL* quality of life, *TB* tuberculosis

### Model Development

**Figure 2:**
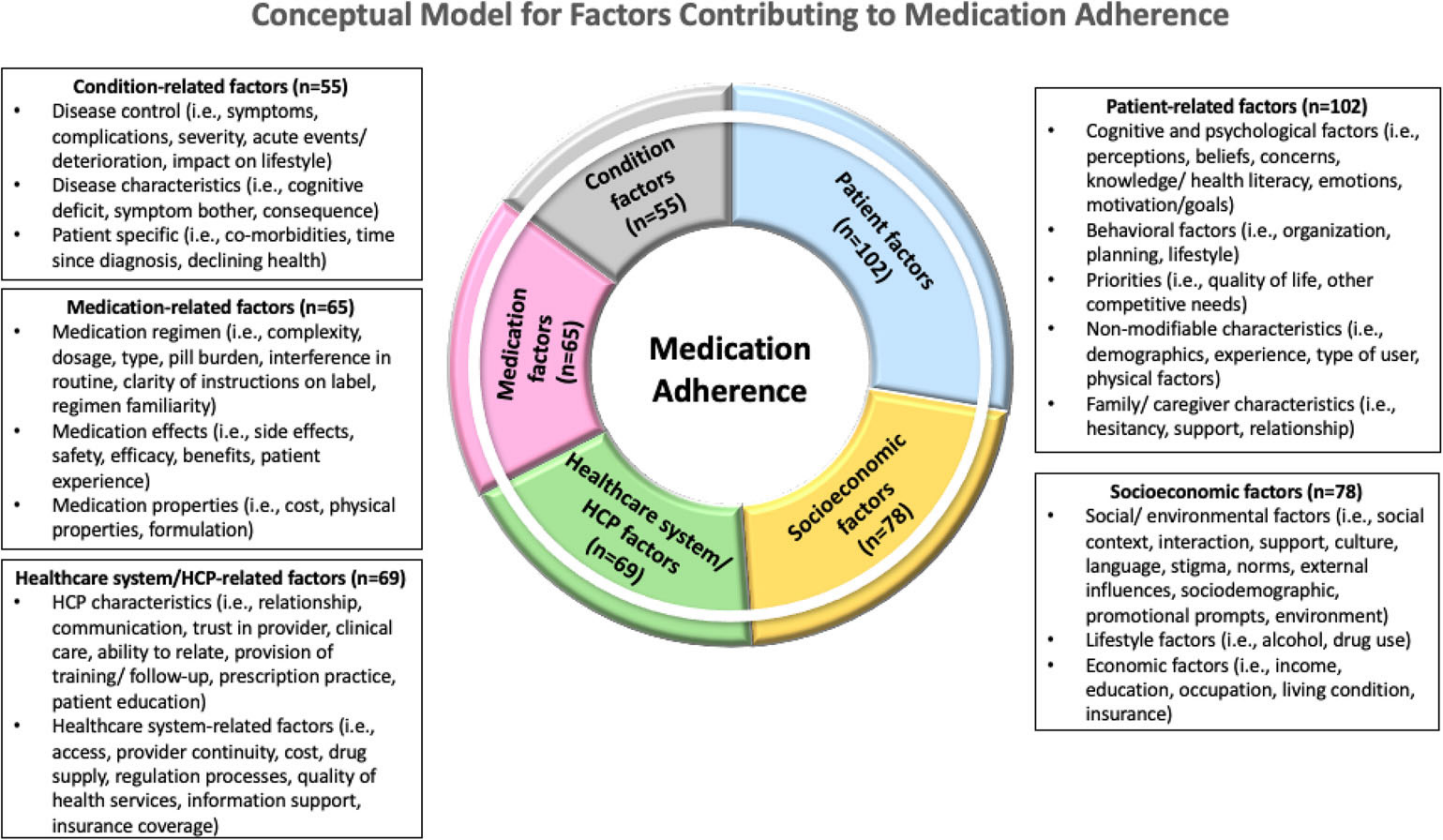
Conceptual model for factors contributing to medication adherence based on a systematic review of 102 conceptual frameworks. Abbreviations: healthcare provider (HCP).

Only 30 models addressed all five dimensions of medication adherence. However, the factors within each model mapped well to one or more of the WHO dimensions, supporting the WHO dimensions to various degrees. Hence, we refined the WHO model and present a “donut model” that (i) provides a succinct overview of the key factors contributing to medication adherence and is adaptable to different patient groups (Fig. 2), (ii) allows readers to appreciate the interconnectivity among the WHO dimensions, and (iii) gains insights into the relative frequency of each dimension for different patient groups. The relative sizes of each slice of the donut correspond to the relative number of studies that support the respective dimensions. The dimensions were arranged in descending order of frequency in the clockwise direction, beginning with patient-related factors, for ease of identification of the most common factors contributing to medication adherence in each patient group. We further categorized the individual factors contributing to medication adherence into sub-themes, as illustrated in the models. A white rim surrounding the five dimensions of medication adherence serves to signify the interconnectivity between the dimensions, an important aspect of medication adherence supported by a number of conceptual frameworks^11, 42, 66, 85^ but were missing or not fully considered in others.^50, 69, 115^

We applied our model to the eight patient groups identified in our systematic review (Supplementary Figures 1a–1i) to describe the factors contributing to medication adherence in these patient groups.

## DISCUSSION

We categorized the available conceptual models by eight patient groups since the key factors contributing to medication adherence can vary depending on the age and type of disease conditions studied. This categorization is reflected by the varying support for each of the WHO dimensions across patient groups.

The common factors across all patient groups were patient-related. Specifically, cognitive and psychological factors such as patients’ beliefs, perceptions, and concerns were most commonly cited. On the other hand, medication-, condition-, healthcare system/HCP-related factors, and socioeconomic factors were less consistently included.

For adults with chronic, non-communicable conditions such as hypertension, hyperlipidemia, diabetes mellitus, patient-related factors, and healthcare system/HCP-related factors were most common. Many patients with the abovementioned chronic conditions are often asymptomatic. The rationale for adhering to medications for non-communicable conditions is often for secondary prevention rather than symptomatic relief. As such, we would expect medication adherence to be dependent on patients’ perceived needs and beliefs about the medication, which are in turn influenced by information and counselling provided by the healthcare provider during the medical encounter.

In adults with symptomatic conditions such as nocturia and migraine, the rationale for adhering to medications would be for symptomatic relief. The extent of symptoms bother and the efficacy of medications in providing symptomatic relief would therefore contribute to patients’ decision to adhere or not adhere to their medications. For adults with psychiatric conditions, patient- and medication-related factors were most commonly cited, likely attributed to cognitive factors and the side effects associated with psychiatric medications.

For adults with cancer, patient-, medication-, and healthcare system/HCP-related factors, and socioeconomic factors were most commonly described. Cancer treatments are often perceived as unpleasant side effects.^121^ As such, patients’ perceptions, beliefs, and concerns about the disease and medications, and the tolerability of medication side effects, as well as the availability of social support, are critical in determining adherence behavior. Additionally, healthcare system/HCP providers provide patients with the necessary information, counselling, comfort, and support that contribute to patients’ decision to adhere to their medications.

For adults taking medications for prevention or treatment of communicable conditions such as HIV and tuberculosis, socioeconomic factors emerged as the second most common dimension aside from patient/caregiver-related factors. The stigma associated with the abovementioned communicable conditions and lack of social support can discourage individuals from accessing treatment or retaining in care.^122^ In resource-limited countries, patients may not adhere to medications simply due to the lack of resources and support to do so. In the pediatrics patient group, children often require additional support to adhere to medications due to young age, explaining the emergence of caregiver characteristics and social support as common factors contributing to medication adherence in this patient group.

Our model contributes to the literature in several ways. Our model can be adapted by clinicians and researchers to study the factors contributing to medication adherence in their patient group of interest. The models may also serve as foundation to refine existing assessment tools for medication adherence by incorporating factors that are deemed to be important for the patient group of interest. We provided a holistic and richer analysis of factors of medication adherence specific for each patient group, as well as factors common across all patient groups. Our models can inform the development of interventions and care models that effectively improve medication adherence and patient outcome for the general population as well as specific patient groups.

Our review process excluded conceptual models that did not study medication adherence from the patients’ perspectives,^123, 124^ as accounts of healthcare providers and family members may be prone to bias. We also excluded three conceptual models that studied medication adherence in the clinical trial settings,^125–127^ as clinical trials may have external prompts or influences not present in actual clinical settings. In view of both the heterogeneity of the evidence and the limited guidance on the rating of evidence in this field, the relative quality of each study was not explored. Additionally, reviewing conceptual frameworks is not as amenable to standardized Risk of Bias tools used by Cochrane and others, which were developed to quantify the rigor of individual studies.^128^ While this review speaks to the frequency of support across different WHO dimensions, it cannot accurately assess unpublished studies that may have been more likely to fail to find support for these WHO dimensions. However, the authors find no reason to think that one WHO dimension would be more likely to suffer from publication bias than another WHO dimension. Future studies can consider creating weights for the factors contributing to medication adherence to allow quantitative testing of our model.

## CONCLUSION

Our conceptual models applied to the different patient groups (supplemental figures) can be utilized as a guide for clinicians and researchers in identifying the facilitators and barriers to medication adherence and developing future interventions aimed at improving medication adherence in these patient groups. Our conceptual model in Figure 2 may also be used for patients who do not match the existing patient groups. In doing so, we hope to achieve better medication adherence and control of disease conditions.

## Supplementary Information

ESM 1 (DOC 81 kb)

ESM 2 (DOCX 2.15 mb)

ESM 3 (DOCX 218 kb)

## References

[1] Vrijens B (2012). *A new taxonomy for describing and defining adherence to medications*. Br J Clin Pharmacol.

[2] World Health Organization (2003). *Adherence to Long-Term Therapies: Evidence For Action*.

[3] Gellad WF (2017). *The myths of medication adherence*. Pharmacoepidemiol Drug Saf.

[4] DiMatteo MR (2004). *Variations in Patients' Adherence to Medical Recommendations: A Quantitative Review of 50 Years of Research*. Med Care.

[5] Jackevicius CA, Mamdani M, Tu JV (2002). *Adherence With Statin Therapy in Elderly Patients With and Without Acute Coronary Syndromes*. JAMA.

[6] Haynes RB, McDonald HP, Garg AX (2002). *Helping Patients Follow Prescribed Treatment Clinical Applications*. JAMA.

[7] Walker EA (2006). *Adherence to Preventive Medications*. Diabetes Care.

[8] Jimmy B, Jose J (2011). *Patient medication adherence: measures in daily practice*. Oman Med J.

[9] Boukhechba M (2018). *A Social Cognitive Theory-based Framework for Monitoring Medication Adherence Applied to Endocrine Therapy in Breast Cancer Survivors*. IEEE EMBS Int Conf Biomed Health Inform.

[10] Murray MD (2004). *A conceptual framework to study medication adherence in older adults*. Am J Geriatr Pharmacother.

[11] Oori MJ (2019). *Conceptual Model of Medication Adherence in Older Adults with High Blood Pressure-An Integrative Review of the Literature*. Curr Hypertens Rev.

[12] Jaam M (2018). *A holistic conceptual framework model to describe medication adherence in and guide interventions in diabetes mellitus*. Res Soc Adm Pharm.

[13] Brawley LR, Culos-Reed SN (2000). *Studying adherence to therapeutic regimens: overview, theories, recommendations*. Control Clin Trials.

[14] Holmes EA, Hughes DA, Morrison VL (2014). *Predicting adherence to medications using health psychology theories: a systematic review of 20 years of empirical research*. Value Health.

[15] **David Moher, A.L**., **Jennifer Tetzlaff, Douglas G Altman**, *Preferred reporting items for systematic reviews and meta-analyses: the PRISMA statement.* BMJ, 2009. **339**. PMC3090117 21603045

[16] Fernandez-Lazaro CI (2019). *Adherence to treatment and related factors among patients with chronic conditions in primary care: a cross-sectional study*. BMC Fam Pract.

[17] Herborg H (2008). *Developing a generic, individualised adherence programme for chronic medication users*. Pharm Pract.

[18] Jansà M (2010). *Multidimensional analysis of treatment adherence in patients with multiple chronic conditions. A cross-sectional study in a tertiary hospital*. Patient Educ Couns.

[19] McLoughlin A, Bennett K, Cahir C (2019). *Developing a model of the determinants of medication nonadherence in older community-dwelling patients*. Ann Behav Med.

[20] Lek Y-Y (1995). and G.d. Bishop, *Perceived vulnerability to illness threats: The role of disease type, risk factor perception and attributions*. Psychol Health.

[21] DiMatteo MR, Haskard SL, Fau-Williams KB, Williams SL (2007). *Health beliefs, disease severity, and patient adherence: a meta-analysis*. Med Care.

[22] **Gil-Girbau M, et al.** Reasons for medication non-initiation: A qualitative exploration of the patients' perspective. Res Social Adm Pharm. 2019. 10.1016/j.sapharm.2019.08.002 31402307

[23] Rottman BM (2017). *Medication adherence as a learning process: insights from cognitive psychology*. Health Psychol Rev.

[24] Bailey SC, Oramasionwu CU, Wolf MS (2013). *Rethinking adherence: a health literacy-informed model of medication self-management*. J Health Commun.

[25] Gearing RE (2011). *Reconceptualizing medication adherence: Six phases of dynamic adherence*. Harvard Rev Psychiatry.

[26] Osterberg L, Blaschke T (2005). *Adherence to Medication*. N Engl J Med.

[27] Pound P (2005). *Resisting medicines: a synthesis of qualitative studies of medicine taking*. Soc Sci Med.

[28] Burton RPD, Hudson T (2001). *Achieving individually sustained commitment to treatment through self-constructed models of medical adherence*. Sociol Spectr.

[29] Dowell J, Hudson H (1997). *A qualitative study of medication-taking behaviour in primary care*. Fam Pract.

[30] Heiby EM, Carlson JG (1986). *The health compliance model*. J Compl Health Care.

[31] Eraker SA, Kirscht JP, Becker MH (1984). *Understanding and improving patient compliance*. Ann Intern Med.

[32] Christensen DB (1978). *Drug-taking compliance: A review and synthesis*. Health Serv Res.

[33] **Maffoni M, et al.** Medication adherence in the older adults with chronic multimorbidity: a systematic review of qualitative studies on patient's experience. Eur Geriatr Med. 2020. 10.1007/s41999-020-00313-2 32297271

[34] Naqvi AA (2019). *A qualitative study investigating perceived barriers to medication adherence in chronic illness patients of Karachi, Pakistan*. J Pak Med Assoc.

[35] August KJ, Billimek J (2016). *A theoretical model of how neighborhood factors contribute to medication nonadherence among disadvantaged chronically ill adults*. J Health Psychol.

[36] Yap AF, Thirumoorthy T, Kwan YH (2016). *Systematic review of the barriers affecting medication adherence in older adults*. Geriatr Gerontol Int.

[37] Linsky A, Simon SR, Bokhour B (2015). *Patient perceptions of proactive medication discontinuation*. Patient Educ Couns.

[38] Jabbour E (2012). *Adherence to BCR-ABL Inhibitors: Issues for CML Therapy*. Clinical Lymphoma Myeloma Leuk.

[39] McHorney CA (2009). *The Adherence Estimator: a brief, proximal screener for patient propensity to adhere to prescription medications for chronic disease*. Curr Med Res Opin.

[40] Dolovich L (2008). *Do patients' expectations influence their use of medications? Qualitative study*. Canadian Family Phys.

[41] Chen CH (2007). *A model of medication-taking behavior in elderly individuals with chronic disease*. J Cardiovasc Nurs.

[42] Piette JD (2006). *A conceptually based approach to understanding chronically ill patients' responses to medication cost pressures*. Soc Sci Med.

[43] Barber N, Safdar A, Franklin BD (2005). *Can human error theory explain non-adherence?*. Pharm World Sci.

[44] Easthall C, Taylor N, Bhattacharya D (2019). *Barriers to medication adherence in patients prescribed medicines for the prevention of cardiovascular disease: a conceptual framework*. Int J Pharm Pract.

[45] Koh JJK (2018). *Access and adherence to medications for the primary and secondary prevention of atherosclerotic cardiovascular disease in Singapore: a qualitative study*. Patient Prefer Adher.

[46] **Petrovic K, Blank TO**. The Andersen-Newman Behavioral Model of Health Service Use as a conceptual basis for understanding patient behavior within the patient-physician dyad: The influence of trust on adherence to statins in older people living with HIV and cardiovascular disease*.* Cogent Psychology, 2015. 2(1).

[47] Spanjol J (2015). *Co-production of prolonged, complex, and negative services: An examination of medication adherence in chronically ill individuals*. J Serv Res.

[48] Brown TM (2012). *Development of a conceptual model of adherence to oral anticoagulants to reduce risk of stroke in patients with atrial fibrillation*. J Manag Care Pharm.

[49] Li WW, Stotts NA, Froelicher ES (2007). *Compliance with antihypertensive medication in Chinese immigrants: cultural specific issues and theoretical application*. Res Theory Nurs Pract.

[50] Li WW (2005). *Cultural factors and medication compliance in Chinese immigrants who are taking antihypertensive medications: instrument development*. J Nurs Meas.

[51] Johnson MJ (2002). *The Medication Adherence Model: a guide for assessing medication taking*. Res Theory Nurs Pract.

[52] Yeam CT (2018). *A systematic review of factors affecting medication adherence among patients with osteoporosis*. Osteoporos Int.

[53] Wozniak LA (2017). *Understanding fragility fracture patients' decision-making process regarding bisphosphonate treatment*. Osteoporos Int.

[54] Brod M, Rousculp M, Cameron A (2008). *Understanding compliance issues for daily self-injectable treatment in ambulatory care settings*. Patient Prefer Adher.

[55] Widayanti AW (2020). *Medicine taking behaviours of people with type 2 diabetes in Indonesia: a qualitative study*. Int J Clin Pharm.

[56] Bockwoldt D (2017). *Understanding Experiences of Diabetes Medications Among African Americans Living With Type 2 Diabetes*. J Transcult Nurs.

[57] Hoefnagels JW (2020). *The Perspectives of Adolescents and Young Adults on Adherence to Prophylaxis in Hemophilia: A Qualitative Study*. Patient Prefer Adher.

[58] Schrijvers LH (2015). *Unravelling adherence to prophylaxis in haemophilia: a patients' perspective*. Haemophilia.

[59] Siekmans K (2018). *Barriers and enablers for iron folic acid (IFA) supplementation in pregnant women*. Mater Child Nutr.

[60] Rosa WE (2020). *A concept analysis of analgesic nonadherence for cancer pain in a time of opioid crisis*. Nurs Outlook.

[61] Xu L, Wang A (2019). *Health belief about adjuvant endocrine therapy in premenopausal breast cancer survivors: a qualitative study*. Patient Prefer Adher.

[62] **Lambert, L.K., et al.**, *Understanding adjuvant endocrine therapy persistence in breast Cancer survivors.* BMC Cancer, 2018. 18(1). 10.1186/s12885-018-4644-7 PMC6042363 29996816

[63] Verbrugghe M (2016). *Factors influencing adherence in cancer patients taking oral tyrosine kinase inhibitors: A qualitative study*. Cancer Nurs.

[64] McGrady ME, Brown GA, Pai AL (2016). *Medication adherence decision-making among adolescents and young adults with cancer*. Eur J Oncol Nurs.

[65] McCue DA, Lohr LK, Pick AM (2014). *Improving Adherence to Oral Cancer Therapy in Clinical Practice*. Pharmacotherapy.

[66] Gater A (2012). *Adherence to oral tyrosine kinase inhibitor therapies in chronic myeloid leukemia*. Leuk Res.

[67] **Hall, N.J., et al.**, *Medication beliefs among patients with inflammatory bowel disease who report low quality of life: A qualitative study.* BMC Gastroenterol, 2007. **7**. 10.1186/1471-230X-7-20 PMC1896170 17559670

[68] Moshkovska T (2008). *Qualitative investigation of patient adherence to 5-aminosalicylic acid therapy in patients with ulcerative colitis*. Inflamm Bowel Dis.

[69] Voshaar M (2016). *Barriers and facilitators to disease-modifying antirheumatic drug use in patients with inflammatory rheumatic diseases: a qualitative theory-based study*. BMC Musculoskelet Disord.

[70] Goh H (2017). *A systematic review of the barriers affecting medication adherence in patients with rheumatic diseases*. Rheumatol Int.

[71] Dockerty T, Latham SK, Smith TO (2016). *Why don't patients take their analgesics? A meta-ethnography assessing the perceptions of medication adherence in patients with osteoarthritis*. Rheumatol Int.

[72] Horne R (2006). *Compliance, Adherence, and Concordance: Implications for Asthma Treatment*. CHEST.

[73] Katić BJ (2010). *Adherence to acute migraine medication: What does it mean, why does it matter?*. Headache.

[74] Jayadevappa R (2015). *Medication adherence in the management of nocturia: Challenges and solutions*. Patient Prefer Adher.

[75] Eshun-Wilson I (2019). *Being HIV positive and staying on antiretroviral therapy in Africa: A qualitative systematic review and theoretical model*. PLoS One.

[76] Graham SM (2018). *HIV care engagement and ART adherence among Kenyan gay, bisexual, and other men who have sex with men: a multi-level model informed by qualitative research*. AIDS Care.

[77] Gill MM (2017). *Understanding Antiretroviral Treatment Adherence Among HIV-Positive Women at Four Postpartum Time Intervals: Qualitative Results from the Kabeho Study in Rwanda*. AIDS Patient Care STDs.

[78] Skovdal M (2011). *Contextual and psychosocial influences on antiretroviral therapy adherence in rural Zimbabwe: towards a systematic framework for programme planners*. Int J Health Plann Manag.

[79] Merten S (2010). *Patient-reported barriers and drivers of adherence to antiretrovirals in sub-Saharan Africa: a meta-ethnography*. Tropical Med Int Health.

[80] Watt MH (2009). *"It's all the time in my mind": facilitators of adherence to antiretroviral therapy in a Tanzanian setting*. Soc Sci Med.

[81] Nam SL (2008). *The relationship of acceptance or denial of HIV-status to antiretroviral adherence among adult HIV patients in urban Botswana*. Soc Sci Med.

[82] Diefenbach-Elstob T (2017). *The social determinants of tuberculosis treatment adherence in a remote region of Papua New Guinea*. BMC Public Health.

[83] van den Boogaard J (2012). *An exploration of patient perceptions of adherence to tuberculosis treatment in Tanzania*. Qual Health Res.

[84] Ho SS, Stenhouse R, Holloway A (2020). *Understanding HIV-positive drug users' experiences of taking highly active antiretroviral treatment: Identity-Values-Conscious engagement model*. J Clin Nurs.

[85] Engler K (2018). *Barriers to antiretroviral therapy adherence in developed countries: a qualitative synthesis to develop a conceptual framework for a new patient-reported outcome measure*. AIDS Care - Psychol Socio-Med Aspects AIDS/HIV.

[86] Fields EL (2017). *Qualitative Comparison of Barriers to Antiretroviral Medication Adherence Among Perinatally and Behaviorally HIV-Infected Youth*. Qual Health Res.

[87] Dima AL (2013). *The Information-Motivation-Behavioral Skills Model of ART adherence in Romanian young adults*. J HIV/AIDS Soc Serv.

[88] Rongkavilit C (2010). *Applying the information-motivation-behavioral skills model in medication adherence among Thai youth living with HIV: a qualitative study*. AIDS Patient Care STDs.

[89] Beusterien KM (2008). *HIV patient insight on adhering to medication: A qualitative analysis*. AIDS Care - Psychol Socio-Med Aspects AIDS/HIV.

[90] Starks H (2008). *Conceptualizing antiretroviral adherence in Beijing*. China AIDS Care.

[91] Fisher JD (2006). *An information-motivation-behavioral skills model of adherence to antiretroviral therapy*. Health Psychol.

[92] Reynolds NR (2003). *The problem of antiretroviral adherence: a self-regulatory model for intervention*. AIDS Care.

[93] Wilson HS, Hutchinson SA, Holzemer WL (2002). *Reconciling incompatibilities: a grounded theory of HIV medication adherence and symptom management*. Qual Health Res.

[94] Barnhoorn F, Adriaanse H (1992). *In search of factors responsible for noncompliance among tuberculosis patients in Wardha District, India*. Soc Sci Med.

[95] Krentel A, Aunger R (2012). *Causal chain mapping: a novel method to analyse treatment compliance decisions relating to lymphatic filariasis elimination in Alor, Indonesia*. Health Policy Plan.

[96] Dubov A, Altice FL, Fraenkel L (2018). *An Information-Motivation-Behavioral Skills Model of PrEP Uptake*. AIDS Behav.

[97] Jacobson KB (2017). *"It's about my life": facilitators of and barriers to isoniazid preventive therapy completion among people living with HIV in rural South Africa*. AIDS Care.

[98] Lim RH, Sharmeen T (2018). *Medicines management issues in dementia and coping strategies used by people living with dementia and family carers: A systematic review*. Int J Geriatr Psychiatry.

[99] **Kikkert, M.J. and J. Dekker**, *Medication Adherence Decisions in Patients With Schizophrenia.* Prim Care Companion CNS Disord, 2017. **19**(6). 10.4088/PCC.17n02182 29216418

[100] Moitra E, Gaudiano BA (2016). *A psychological flexibility model of medication adherence in psychotic-spectrum disorders*. J Contextual Behav Sci.

[101] O’Callaghan P (2014). *Adherence to stimulants in adult ADHD*. ADHD Attention Deficit Hyperact Disorders.

[102] Gault I, Gallagher A, Chambers M (2013). *Perspectives on medicine adherence in service users and carers with experience of legally sanctioned detention and medication: a qualitative study*. Patient Prefer Adher.

[103] Sanders JJ (2013). *Meaning and methadone: Patient perceptions of methadone dose and a model to promote adherence to maintenance treatment*. J Addict Med.

[104] Hon A (2012). *Factors influencing the adherence of antipsychotic medication (Aripiprazole) in first-episode psychosis: findings from a grounded theory study*. J Psychiatr Ment Health Nurs.

[105] Roe D (2009). *Why and how people decide to stop taking prescribed psychiatric medication: exploring the subjective process of choice*. Psychiatric Rehabil J.

[106] McCann TV, Clark E, Lu S (2008). *The self-efficacy model of medication adherence in chronic mental illness*. J Clin Nurs.

[107] Corrigan PW (2002). *Adherence to anti-psychotic medications and Health Behavior theories*. J Ment Health.

[108] Perkins DO (1999). *Adherence to antipsychotic medications*. J Clin Psychiatry.

[109] Fenton WS, Blyler CR, Heinssen RK (1997). *Determinants of medication compliance in schizophrenia: empirical and clinical findings*. Schizophr Bull.

[110] Davidhizar R (1984). *The schizophrenic client's reinforcement and punishment for medication adherence*. Issues Mental Health Nurs.

[111] Jamison KR, Akiskal HS (1983). *Medication compliance in patients with bipolar disorder*. Psychiatr Clin N Am.

[112] Nebot Giralt A (2019). *Understanding acceptance of and adherence to a new formulation of paediatric antiretroviral treatment in the form of pellets (LPV/r)-A realist evaluation*. PLoS One.

[113] Galea JT (2018). *Barriers and facilitators to antiretroviral therapy adherence among Peruvian adolescents living with HIV: A qualitative study*. PLoS One.

[114] Olds P (2015). *Explaining Antiretroviral Therapy Adherence Success Among HIV-Infected Children in Rural Uganda: A Qualitative Study*. AIDS Behav.

[115] Haberer J, Mellins C (2009). *Pediatric adherence to HIV antiretroviral therapy*. Curr HIV/AIDS Rep.

[116] Vreeman RC (2009). *Factors sustaining pediatric adherence to antiretroviral therapy in western Kenya*. Qual Health Res.

[117] Sonney JT, Insel KC (2016). *Reformulating the Common Sense Model of Self-Regulation: Toward Parent-Child Shared Regulation*. Nurs Sci Q.

[118] Goh XT (2017). *A systematic review of factors that influence treatment adherence in paediatric oncology patients*. J Clin Pharm Ther.

[119] Heneghan MB (2020). *Applying the COM-B model to patient-reported barriers to medication adherence in pediatric acute lymphoblastic leukemia*. Pediatr Blood Cancer.

[120] Khan MU, Aslani P (2019). *A Review of Factors Influencing the Three Phases of Medication Adherence in People with Attention-Deficit/Hyperactivity Disorder*. J Child Adolesc Psychopharm.

[121] Robb KA (2014). *Public perceptions of cancer: a qualitative study of the balance of positive and negative beliefs*. BMJ Open.

[122] Rintamaki LS (2006). *Social stigma concerns and HIV medication adherence*. AIDS Patient Care STDs.

[123] Vedana KG (2016). *Meaning of Pharmacological Treatment for Families of People With Depression*. Issues Ment Health Nurs.

[124] Heidari P (2019). *Rheumatologists' insight into medication adherence in patients with rheumatoid arthritis: A qualitative study*. Int J Rheum Dis.

[125] Ickovics JR, Meisler AW (1997). *Adherence in AIDS clinical trials: a framework for clinical research and clinical care*. J Clin Epidemiol.

[126] Corneli A (2016). *Participants' Explanations for Nonadherence in the FEM-PrEP Clinical Trial*. J Acquir Immune Defic Syndr.

[127] Ferrer RA (2010). *Toward an information-motivation-behavioral skills model of microbicide adherence in clinical trials*. AIDS Care.

[128] Armijo-Olivo S (2012). *Assessment of study quality for systematic reviews: a comparison of the Cochrane Collaboration Risk of Bias Tool and the Effective Public Health Practice Project Quality Assessment Tool: methodological research*. J Eval Clin Pract.

