# Supplementary material for: An Adaptable Framework for Factors Contributing to Medication Adherence: Results from a Systematic Review of 102 Conceptual Frameworks
Source: J Gen Intern Med. 2021 Mar 3;36(9):2784–95. doi: 10.1007/s11606-021-06648-1 (PMC8390603; doi:10.1007/s11606-021-06648-1)
Supplement: Supplementary file 2 — (DOCX 2.15 mb) [file 11606_2021_6648_MOESM2_ESM.docx]

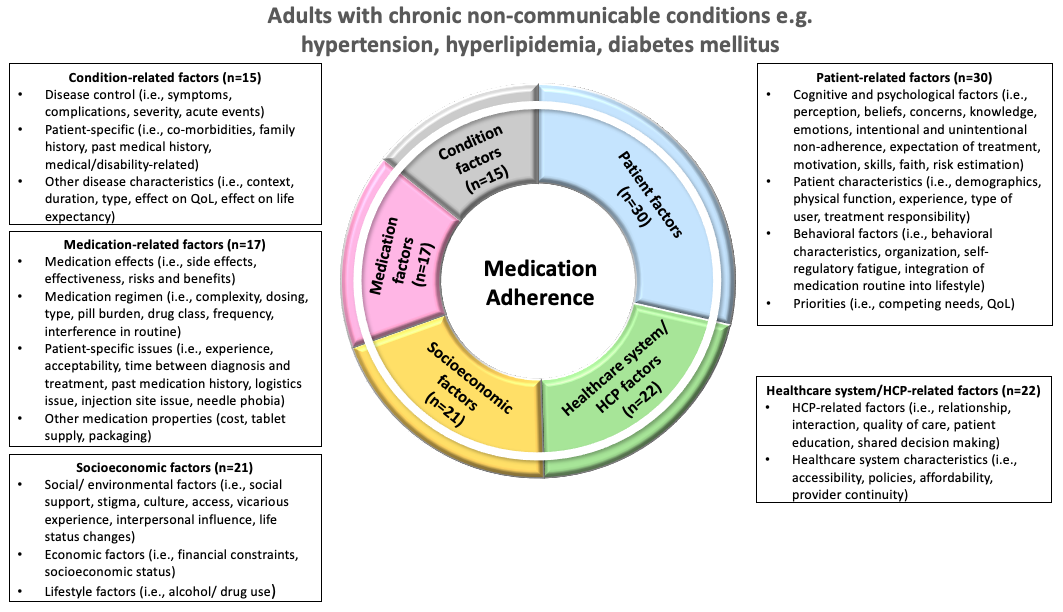


**Supplementary Figure 1a:** Conceptual model for factors contributing to medication adherence in adults with chronic, non-communicable conditions such as cardiovascular diseases, diabetes mellitus, hemophilia, osteoporosis and anemia secondary to pregnancy. Abbreviations: healthcare provider (HCP); quality of life (QoL)

**
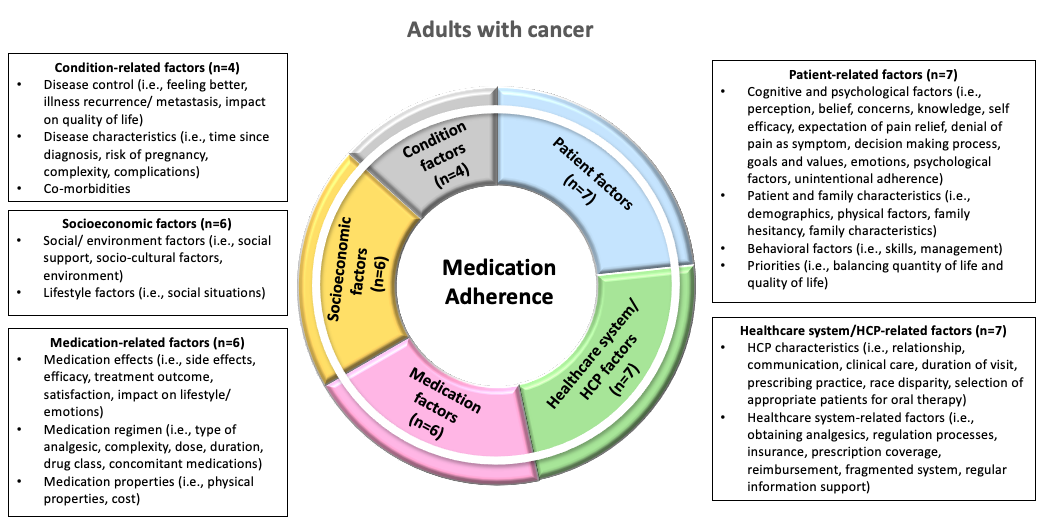
**

**Supplementary Figure 1b:** Conceptual model for factors contributing to medication adherence in adults with cancer. Abbreviations: healthcare provider (HCP)

**
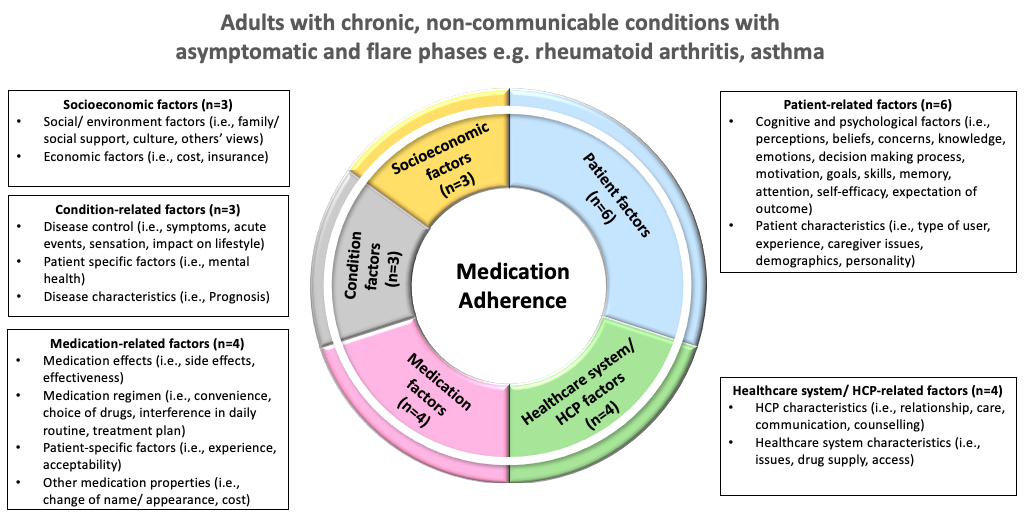
**

**Supplementary Figure 1c:** Conceptual model for factors contributing to medication adherence in adults with chronic, non-communicable conditions with asymptomatic and flare phases e.g. rheumatoid arthritis and asthma. Abbreviations: healthcare provider (HCP)

**
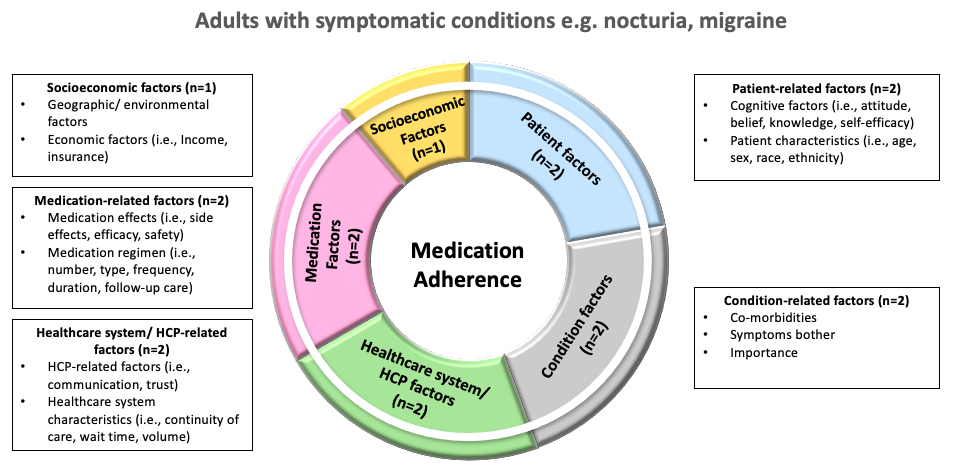
**

**Supplementary Figure 1d:** Conceptual model for factors contributing to medication adherence in adults with symptomatic conditions such as nocturia and migraine. Abbreviations: healthcare provider (HCP)

**
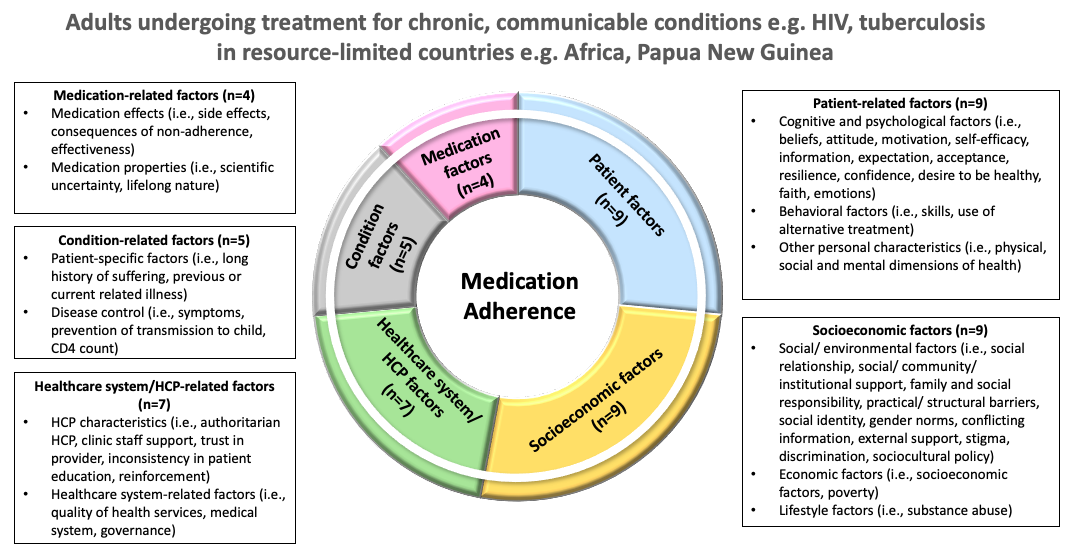
**

**Supplementary Figure 1e:** Conceptual model for factors contributing to medication adherence in adults undergoing treatment for chronic, communicable conditions e.g. HIV, tuberculosis in resource-limited countries e.g. Africa, Papua New Guinea. Abbreviations: healthcare provider (HCP); human immunodeficiency virus (HIV)

**
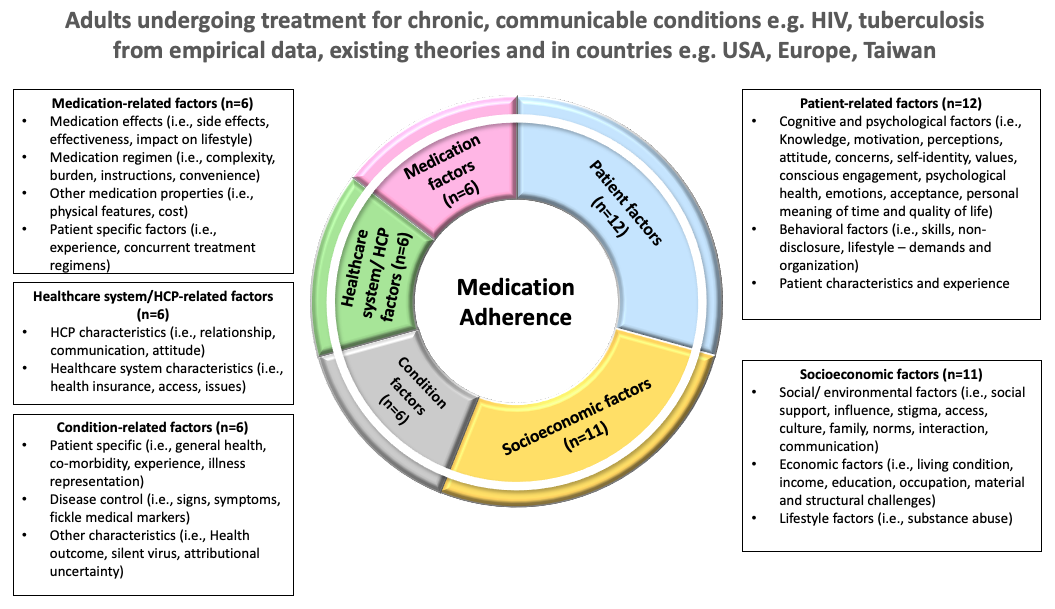
**

**Supplementary Figure 1f:** Conceptual model for factors contributing to medication adherence in adults undergoing treatment for chronic, communicable conditions e.g. HIV, tuberculosis from empirical data, existing theories and in countries e.g. USA, Europe, Taiwan. Abbreviations: healthcare provider (HCP), human immunodeficiency virus (HIV); United States of America (USA)

**
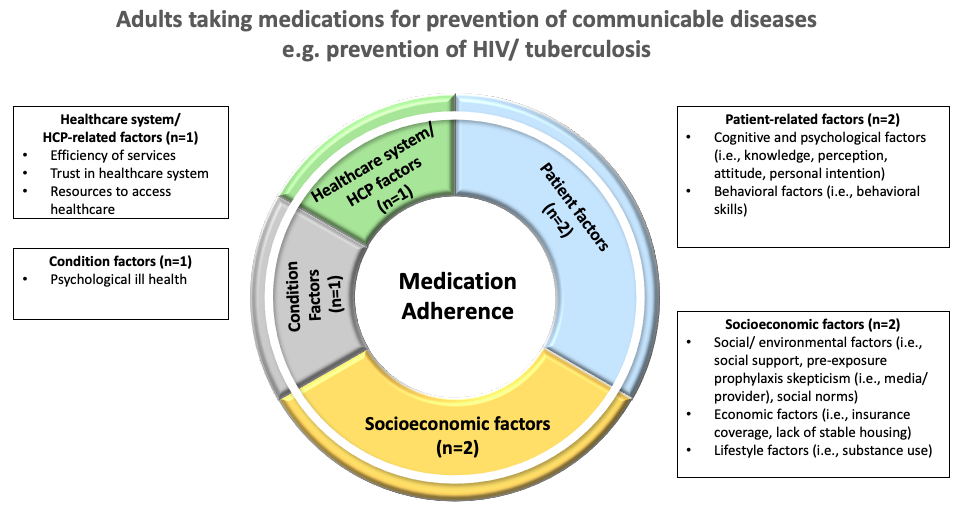
**

**Supplementary Figure 1g:** Conceptual model for factors contributing to medication adherence in adults taking medications for prevention of communicable conditions such as HIV and tuberculosis. Abbreviations: healthcare provider (HCP); human immunodeficiency virus (HIV)

**
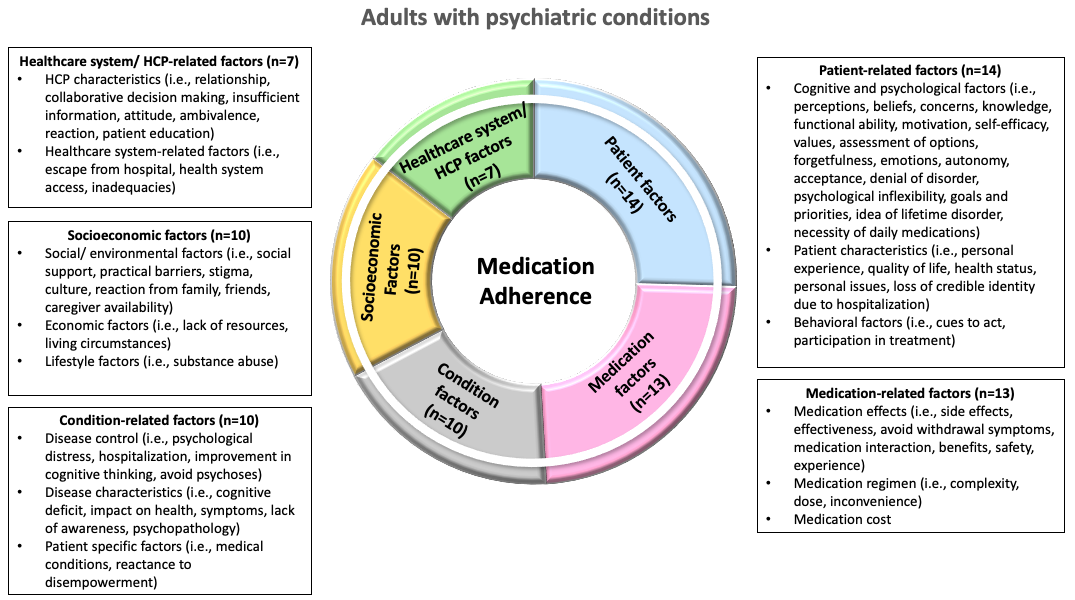
**

**Supplementary Figure 1h:** Conceptual model for factors contributing to medication adherence in adults with psychiatric conditions. Abbreviations: healthcare provider (HCP)

**
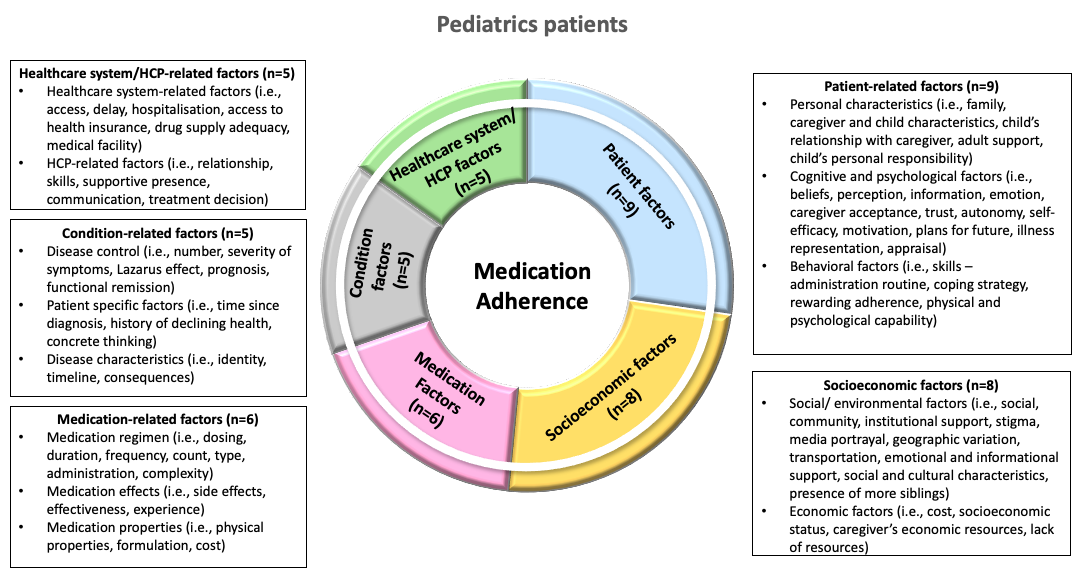
**

**Supplementary Figure 1i:** Conceptual model for factors contributing to medication adherence in pediatrics patients. Abbreviations: healthcare provider (HCP)
