# Supplementary material for: An Adaptable Framework for Factors Contributing to Medication Adherence: Results from a Systematic Review of 102 Conceptual Frameworks
Source: J Gen Intern Med. 2021 Mar 3;36(9):2784–95. doi: 10.1007/s11606-021-06648-1 (PMC8390603; doi:10.1007/s11606-021-06648-1)
Supplement: Supplementary file 3 — (DOCX 218 kb) [file 11606_2021_6648_MOESM3_ESM.docx]

**Supplementary Table 1: Search strategy for Embase**

|  | Search strategy |
| --- | --- |
| 1 | 'patient compliance'/de OR 'medication compliance'/exp |
| 2 | ((patient OR drug OR medication OR therap* OR treatment* OR pharmacotherap*) NEAR/3 (complian* OR adheren* OR noncomplian* OR nonadheren* OR persisten*)):ab,ti |
| 3 | 'conceptual model'/exp OR 'theoretical model'/exp |
| 4 | framework*:ab,ti OR model*:ab,ti OR theor*:ab,ti OR concept*:ab,ti |
| 5 | (#1 OR #2) AND (#3 OR #4) |

**Supplementary Table 2: Search strategy for CINAHL**

| Search strategy | |
| --- | --- |
| S1 | (MH "Patient Compliance+") |
| S2 | TI (patient OR drug OR medication OR therap* OR treatment* OR pharmacotherap*) N3 (complian* OR adheren* OR noncomplian* OR nonadheren* OR persisten*) |
| S3 | AB (patient OR drug OR medication OR therap* OR treatment* OR pharmacotherap*) N3 (complian* OR adheren* OR noncomplian* OR nonadheren* OR persisten*) |
| S4 | MH (Models, theoretical OR conceptual framework) |
| S5 | TI (framework* OR model* OR theor* OR concept*) |
| S6 | AB (framework* OR model* OR theor* OR concept*) |
| S7 | (S1 OR S2 OR S3) AND (S4 OR S5 OR S6) |

**Supplementary Table 3: Search strategy for PsycINFO**

|  | Search strategy |  |
| --- | --- | --- |
| 1 | exp treatment compliance/ |  |
| 2 | ((patient or drug or medication or therap* or treatment* or pharmacotherap*) adj3 (complian* or adheren* or noncomplian* or nonadheren* or persisten*)).ab,ti. |  |
| 3 | exp models/ |  |
| 4 | (framework* or model* or theor* or concept*).ab,ti. |  |
| 5 | (1 OR 2) AND (3 OR 4) |  |

**Supplementary Table 4: Search strategy for PubMed**

|  | Search strategy |  |
| --- | --- | --- |
| 1 | patient compliance[MeSH] |  |
| 2 | (patient[tiab] OR drug[tiab] OR medication[tiab] OR therap*[tiab] OR treatment*[tiab] OR pharmacotherap*[tiab]) AND (complian*[tiab] OR adheren*[tiab] OR nonadheren*[tiab] OR noncomplian*[tiab] OR persisten*[tiab]) |  |
| 3 | “Models, Theoretical”[Mesh] |  |
| 4 | **framework*[tiab] OR model*[tiab] OR theor*[tiab] OR concept*[tiab]** |  |
| 5 | (#1 OR #2) AND (#3 OR #4) |  |

**Supplementary Table 5: Summary of conceptual frameworks for factors contributing to medication adherence not specific to any patient group. Abbreviations: Healthcare provider (HCP)**

| **Author, Publication Year** | **Model Purpose** | **Patient/ caregiver-related factors in model** | **Condition-related factors in model** | **Medication-related factors in model** | **Healthcare system/ HCP-related factors** | **Socioeconomic factors** | **Comments** |
| --- | --- | --- | --- | --- | --- | --- | --- |
| Conceptual frameworks **not specific to any patient group** | | | | | | | |
| Gil-Girbau, et al. 2019 [22] | To identify reasons for medication non-initiation among primary care patients with distinct treatment profiles | Health literacy; Emotional response; Perception of Disease;  Medication perception | Embedded in perception of disease | Embedded in perception of medication | GP-patient relationship; other health professionals | Social and cultural factors; external influences (interpersonal, contextual factors) | Focuses on medication non-initiation; with perception of risk-benefit balance being the central focus. Does not include specialised care, dependent pediatric or geriatric populations |
| Rottman, et al. 2017 [23] | Clinicians can more effectively address patients’ misconceptions and biases, helping the patient develop accurate impressions of the medication | Beliefs | Not explicitly included in model | Experience (e.g. side effects) | Not explicitly included in model | Not explicitly included in model | Conceptualises non-adherence as a causal-learning process; focuses on integration of personal experiences, beliefs about medications and adherence to form a cyclic learning problem |
| Bailey, et al. 2013 [24] | To review, criticize current adherence measures and to offer guidance to future interventions promoting medication self-management | Understanding; knowledge; awareness; fear of side effects; lifestyle; organization and planning; forgetful; loss of interest; conserve supply and reduce costs | Lack of symptoms | Drug cost; side effects; unclear label instructions; lack of regimen familiarity | Provider-patient relationship | Not explicitly included in model | Presents model of medication self-management in terms of a cyclic process involving fill, understand, organise, take, monitor, sustain. |
| Gearing, et al. 2011 [25] | Introduce dynamic adherence, a six-phase model, which incorporates the role of transactional processes and other factors that influence patients’ adherence decisions | Prospect Theory; time-preference and health capital theories | Not explicitly included in model | Not explicitly included in model | Bilateral-bargaining theory | Consumer choice theory | Focuses on relational and economic factors that includes individual characteristics, patient-provider relationship, and cost-benefit implications of patients’ treatment decisions. Limited attention to condition- and medication-related factors. |
| Osterberg, et al. 2005 [26] | To improve adherence to medication regimen | Patient provider communication; patient’s interaction with healthcare system | Not explicitly included in model | Physician prescribes overly complex regimen | Patient provider communication; patient’s interaction with healthcare system; physician’s interaction with healthcare system | Not explicitly included in model | Emphasises interaction of patient, provider and the healthcare system; limited attention to condition-, medication- and socio-economic factors. |
| Pound, et al.2005 [27] | To synthesise qualitative studies of lay experiences of medicine taking | Worries and concerns about medicine; evaluation of medication; type of medication user - passive accepter/ active acceptors/ active modifiers/ rejectors | Not explicitly included in model | Embedded in patients’ worries and concerns about medicine | Not explicitly included in model | Not explicitly included in model | Concludes that the main reason for medication non-adherence is not because of failings in patients, doctors or systems, but because of concerns about the medicines |
| Burton, et al. 2001 [28] | To show that self-construction theories can address major shortcomings of traditional medical and patient models used in adherence research | Intensive affective commitment, extensive affective commitment,  cognitive commitment,  cognitive activity;  Identity salience; role meaning | Not explicitly included in model | Not explicitly included in model | Not explicitly included in model | Social context | Focuses on individual’s social and intrapersonal construction of self; does not provide for a more critical/ materialistic understanding of issues surrounding use of medications in treating illness |
| Dowell, et al. 1997 [29] | To investigate the factors which influence medication-taking behavior in primary care | Knowledge; faith in the doctor; motivation to start medicines; type of medicine user; acceptance of illness; | Symptoms | Benefits and perceived drawbacks | Doctor’s advice | Practical problems | Presents a model illustrating the dynamic decision-making process; focuses on patients’ perceptions. |
| Heiby, et al. 1986 [30] | To integrate the effects of both the immediate and the delayed consequences of (non-)compliance with the moderating aspects of its cognitive constructs | Perceptions; Beliefs and attitudes | Not explicitly included in model | Drug dosage and characteristics; instructions; consequences (reinforcement, benefit, cost, punishment) | Subject-provider interaction | Social support - family, friends, therapeutic groups; promotional – media and education, pharmacist, reminders; optimal quantity and frequency of prompts | Presents a cognitive-behavioral model of compliance with emphasis on taking daily medicines; |
| Eraker, et al. 1984 [31] | provides a framework for modifying general health beliefs; treatment recommendations; experience with therapeutic regimens and HCP; patient knowledge; social  interaction patterns | General and specific health beliefs; patients’ preferences; knowledge; experience; sociodemographics; health decision | Health outcome; experience with disease | Experience with diagnostic and therapeutic interventions | Patient-physician relationship | Social interaction; sociodemographics | Presents a health decision model focuses on health beliefs and patients’ preferences, including decision analysis and behavioral decision theory |
| Christensen 1978 [32] | For developing intervention strategies to improve compliance | Attitudes, beliefs, perceptions, expectations, Assessment of the seriousness of his condition and the benefits and costs of alternative actions | Recurrence of symptoms | Cues – experiences, side effects, costs, partial or complete relief of symptoms | Physician-patient relationship, physician’s ability to relate, provision of information to patient | Background factors - level of education, and peer group norms. | Modified Health Belief model, adopts the perspective of the patient who constantly reassesses the decision to comply (and extent of compliance) with prescribed instructions as he seeks medical help and proceeds through convalescence |

**Supplementary Table 6: Summary of conceptual frameworks for factors contributing to medication adherence in adults with chronic, non-communicable conditions e.g. hypertension, hyperlipidemia, diabetes mellitus. Abbreviations: Ascertaining Barriers to Compliance (ABC); Adolescents and Young Adults (AYAs); Healthcare provider (HCP); Human Immunodeficiency Virus (HIV); World Health Organisation (WHO)**

| **Author, Publication Year** | **Model Purpose** | **Patient/ caregiver-related factors in model** | **Condition-related factors in model** | **Medication-related factors in model** | **Healthcare system/ HCP-related factors** | **Socioeconomic factors** | **Comments** |
| --- | --- | --- | --- | --- | --- | --- | --- |
| Adults with **chronic, non-communicable conditions** e.g. hypertension, hyperlipidemia, diabetes mellitus | | | | | | | |
| Hoefnagels, et al. 2020 [57] | To explore the underlying reasons for (non)adherence to prophylaxis in haemophilia from the perspective of AYAs | Varying treatment responsibilities; risk estimation | Embedded in risk estimation | Not explicitly included in model | Not explicitly included in model | Not explicitly included in model | Focuses varying treatment responsibilities and risk estimation by AYAs. Does not account for medication, health system and socio-economic factors. |
| Maffoni, et al. 2020 [33] | Generate a patient’s decisional flowchart, to sum up possible behavioral outcomes of the adherence process | Patient’s experience, capabilities; Health literacy; decision to obtain and to take medicines; intentional or non-intentional (e.g. forgetfulness); beliefs and concerns (about treatment, polypharmacy, drug prioritisation) | Not explicitly included in model | Treatment characteristics and complexity | Prescriber-patient relationship | Family and social support | integrated barriers and facilitators retraced within the framework of the ABC Taxonomy model to generate a patient’s decisional flowchart; condition-related factors not outlined. |
| Naqvi, et al. 2019 [34] | To examine perceived barriers to medication adherence in patients with chronic illnesses | Forgetfulness; feel good without medicine; knowledge about drug therapy; low perceived value of medicine | Co-morbidities | Adverse drug reaction; regimen complexity; pill burden | Not explicitly included in model | Financial constraints | Examines intentional, non-intentional and cost-related non-adherence; does not include healthcare system/ HCP factors. |
| Oori, et al. 2019 [11] | Develop a model based on the ecological approach to predict medication adherence in older adults with high blood pressure | Behavioral characteristics, biological, psychological characteristics, level of personal knowledge; patient-family relationship; | Disease control, family history, duration, history of fall, co-morbidities | Number of medications, duration, cost of medication, interference in routine, medication adherence effects | Patient-HCP relationship, Healthcare organisation characteristics or practice patterns, supervising programs | Social factors at individual level, community health policies | Suggests direct influence of personal, interpersonal, health system, social factors and indirect influence of environmental factors on adherence; effects of adherence influence personal factors as feedback effect. |
| Widayanti, et al. 2019 [55] | Provide insight into the medicine-taking behaviors of disadvantaged people in Indonesia | Type of user - Passive acceptors, active acceptors, active modifier, rejectors; self-evaluation of medications | Symptoms | Effectiveness, adverse effects, acceptability and cost of medicine compared to traditional medicines | Not explicitly included in model | Not explicitly included in model |  |
| Easthall, et al. 2018 [44] | Produce a conceptual framework for developing a questionnaire-based medication adherence tool. | Knowledge, skills, beliefs about capabilities and consequences, motivation and goals, goal conflicts, memory, attention, decision processes, emotion | Not explicitly included in model | Not explicitly included in model | Environmental context and resources, social influences | Environmental context and resources, social influences | Adapted from Theoretical Domains Framework with added domain of ‘goal conflict’; does not account for condition- and medication-related factors. |
| Jaam, et al. 2018 [12] | Develop a conceptual model for future research and interventions targeting medication adherence in patients with diabetes | Demographics, knowledge, psychological feelings, quality of life, beliefs and perceptions, other factors | Co-morbidities Asymptomatic disease, type of diabetes, disease complications, disease duration, glycemic control | Complexity of regimen, drug class, adverse effects, lifestyle-related changes, previous experience | Patient-provider interactions, provider perception, knowledge, workload,  Financial, resources, care process | Support, vicarious experience, stigma, culture | A comprehensive model summarising the complex network of influencing factors and describes relations between factors. |
| Koh, et al. 2018 [45] | Propose a framework to broaden insight into factors of medication adherence | Ability to perceive, ability to seek, ability to reach, ability to pay, ability to engage; | Perception of seriousness of condition | Difficulty in managing medication regimen, side effects | Approachability, acceptability, availability, affordability, appropriateness | Integrated with patient factors and health system/ HCP factors | Emphasises medication access and its impact on medication adherence; limited attention to condition and medication factors |
| Siekmans, et al. 2018 [59] | To inform large scale iron and  folic acid supplementation programme design for pregnant women | Knowledge, attitude, skills, self-efficacy | Not explicitly included in model | Tablet supply and packaging | Resources; Message timing and targeting; quality of care by provider | Social support/ stigma; environment/ access | Guided by socioecological framework and Theory of Triadic influence; outlines factors influencing access and adherence to supplementation. |
| Yeam, et al. 2018 [52] | To aid clinicians in remembering the factors and applying it in their daily clinical practice. | Demographics; physical and mental function; menopausal-related factors; disease and treatment perceptions; others | Past medical history; comorbidities; medical screening for osteoporosis, family history | Dosing regimen; side effects; medication type; past medication history; others | Physician-specific factor; facilities factors; trust in physician; communication and support from HCP; access; policies | Social factors; economic factors; lifestyle factors | Adapted from WHO classification; allow better visualisation of factors influencing adherence. |
| Bockwoldt, et al. 2017 [56] | To describe the experiences of taking diabetes medications among midlife African American men and women with type 2 diabetes and to identify factors that  influence these experiences | Difficulty integrating medication routine into lifestyle; emotions; perceptions; new knowledge; re-appraisal of self and situation; faith | Acute physical events; sensations high/low glucose; glucose readings; target HbA1C | Side effects; needle-phobia | Healthcare system issues; physician cares; Help from diabetes educators | Life status changes; family support | Examines adaptive and ineffective experiences of medication taking; Acute physical events, knowledge, life status changes, reappraisal of self and situation are turning points that lead to behavior change. |
| Wozniak, et al. 2017 [53] | To understand how patients 50 years and older decided to persist with or stop osteoporosis treatment | Reassessment over time | Severity of osteoporosis; future impact | Benefit and risk of prescription treatment | Not explicitly included in model | Not explicitly included in model | Conceptualisation of decision-making process to persist, stop or restart prescription treatment for clinical osteoporosis over time; does not account for health system, social factors |
| August, et al. 2016 [35] | For understanding the mechanisms whereby the neighborhood environment may impact medication nonadherence among individuals most at risk for adverse disease outcomes | Health beliefs; self-efficacy | Disease context | Not explicitly included in model | Not explicitly included in model | Health-related social support/ control; Factors associated with low socio-economic status and disadvantaged neighbourhood are described | Focuses on contribution of neighbourhood factors on medication adherence; takes into account patient and disease factors as potential moderators. Does not account for medication and health system factors. |
| Yap, et al. 2016 [36] | To allow clinicians to remember these factors better, and to apply these factors in their daily encounters with older adults | Demographics; medical history; beliefs/ knowledge; others; mental state; habits/ behavior; physical health; others | Not explicitly included in model | Drug; drug regimen; drug handling; others | Physician- and system factors are described | Not explicitly included in model | Allows better visualisation of factors influencing medication adherence in geriatric population; little attention to condition-related factors |
| Linsky, et al. 2015 [37] | To identify patient perspectives on intentional medication discontinuation in order to optimize medication use | Conflicting views of medication; Limited experience with medication discontinuation | Not explicitly included in model | Not explicitly included in model | Importance of patient-provider relationships - trust, relying on expertise, shared decision making, balancing multiple providers | Not explicitly included in model | Suggests that patients’ views of medication are present prior to, but may be changed by relationship with HCP; and proactive medication discontinuation would not commence prior to interaction with HCP |
| Petrovic, et al. 2015 [46] | To gain insight into the context within which adherence occurs for older people living with HIV and cardiovascular disease | Aging, Demographic variables, personal attitudes, sets of beliefs, trust beliefs; mental health issues; Personal beliefs systems | Not explicitly included in model | Not explicitly included in model | Lack of access to healthcare; trust in patient-doctor relationship | Alcohol use/ abuse | Emphasises influence of trust on adherence; does not account for condition and medication factors |
| Schrijvers, et al. 2015 [58] | to clarify the process underlying adherence to  prophylaxis in severe haemophilia from a patients’ perspective | Acceptance; Understanding; Planning and infusing skills; Perception; ability to exert prophylaxis; position of prophylaxis in life | Feeling and fearing symptoms | Not explicitly included in model | Not explicitly included in model | Not explicitly included in model | Adherence is determined by the position of prophylaxis in life, which is influenced by a combination of perceptions and skills; does not account for medication, health system/HCP and socio-economic factors. |
| Spanjol, et al. 2015 [47] | Develop a novel theoretical framework of adherence as a nested system of coproduction behaviors, characterized by temporal and scope dimensions. | Medication consumption initiation and completion cues; self-regulatory fatigue, organization and maintenance of instrumental medication tools; instability in living environment | Not explicitly included in model | Not explicitly included in model | Multi-step nature/ complexity of medication refill | Not explicitly included in model | Recasts adherence as a system of coproduction behaviors and routines that differ along temporal and scope dimensions; does not account for condition-, medication- and socio-economic factors |
| Brown, et al. 2012 [48] | Identifies an adherence process that can guide opportunities for effective interventions | Knowledge base and reinforcement, motivation, Personalized system, habit formation, and system adaptation, Self-efficacy loop; pre-disposing, moderating, and contextual factors | Not explicitly included in model | Not explicitly included in model | Knowledge base and reinforcement – reinforcing role of healthcare provider | Predisposing, moderating, and contextual factors | Emphasises knowledge, motivation, personal system and a feedback loop that supports self-efficacy and adherence; does not account for condition- and medication-factors |
| Jabbour, et al. 2012 [38] | Discuss issues of oral medication  adherence in chronic conditions in general | Age; psychological/ emotional factors; conscious decision; no understanding of risks and benefits of treatment; health beliefs and expectations of treatment | Not explicitly included in model | Frequency/ complexity of dosing; toxicity/ side effects; immediacy and evidence of benefit; costs; time between diagnosis and treatment | Prescription of complex treatment; Communication/ relationship with patient; Poor patient education; Poor consideration of patient's lifestyle or ability to afford; Low job satisfaction; limited/ inconvenient access to healthcare, high cost of treatment | Not explicitly included in model | Focuses on barriers of medication adherence; does not consider condition- and socioeconomic factors |
| McHorney  2009 [39] | To organise the myriad hypothesised adherence determinants on a conceptual map for methodological work on the Adherence Estimator | Demographics, Generic psychosocial beliefs, states and skills, Disease-related beliefs and skills, Treatment-related beliefs | Not explicitly included in model | Not explicitly included in model | Not explicitly included in model | Not explicitly included in model | Focuses on intentional non-adherence |
| Brod, et al. 2008 [54] | Understanding compliance issues for long term self-injectable treatments, using a chronic condition (osteoporosis) as a model | Motivation: perceived severity, fracture risk; Initial expectations | Not explicitly included in model | Injection site issues; Logistical injection issues; side effects; Efficacy results | Physician knowledge/ understanding of efficacy, clinical profile, ability/time to train patients on self-injectable and handle coverage issues; Physician message; provision of training follow up; | Travel issues (refrigeration, airline security); Cost/ coverage issues | Examines factors influencing adherence at various time points; emphasises patient and physician factors; does not account for condition-related factors. |
| Dolovich, et al. 2008 [40] | To show the linkages between patients’ expectations of their medications and their medication taking behavior | Expectations of taking medications; strategies to confirm/ modify expectations; Contextual factors - Beliefs, previous experiences with medications | Embedded in consequences of adherence | Consequences of adherence; cost of medication | Intervening factors - Relationship with healthcare providers | Intervening factors - Other people’s beliefs | Relates patient’s expectations of their medication to their medication taking behavior; includes contribution of contextual factors, intervening factors and consequences of adherence. |
| Chen, et al. 2007 [41] | For health professionals to design valid interventions for elderly patients to increase medication adherence | Perceived effectiveness; Perceived reality; memory deficit, other competitive needs; tailoring regimen to daily habits, special reminder pill packaging | Deterioration of physical condition | Complex dosage schedules; simplified dosing regimens | Perceived partnership | Interpersonal influences; family support | Presents factors influencing patients’ readiness to adhere and factors that convert perceptions into actions. |
| Li, et al. 2007 [49] | To guide studies of medication compliance in this population and to assist healthcare providers to support compliance with antihypertensive treatments for Chinese immigrants | Sociodemographics; health concern in general; cultural health perception; Cultural healthcare | Comorbidity | Frequency of medications, types of medications | Patient-provider relationship, provider continuity | Socio-demographics; social support (perceived from cultural norms) | Modified Sick-Role Behavior model; focuses on studying independent factors associated with medication compliance, the interplay between factors (e.g., benefits and side effects of medications) are  not discussed |
| Piette, et al. 2006 [42] | For understanding chronically ill patients’ medication cost problems that focuses on out-of-pocket costs as well as additional characteristics of the treatment, patient, provider, and health system | Perceived benefits of treatment; mental status; self-efficacy; health literacy | Effect on current health-related quality of life; Effect on life expectancy | Regimen complexity; regimen; medication characteristics - adverse effects, dosing complexity, perceived need | Knowledge of costs; therapeutic choice fostering trust; Discussion about costs and adherence; referrals to medication cost assistance programs;  Screening for cost problems; barriers to refilling medication/ applying for benefits; prescriber incentives | Sociocultural influences; financial pressures; income; regimen coverage; out-of-pocket medication costs  Other health costs | Focuses on cost-related underuse of medication |
| Barber, et al. 2005 [43] | Offers a new and valuable way of understanding non-adherence, and could inform interventions | Active failure - slips and lapses, mistakes (wrong plans), violations; Error producing conditions - disruption of routine, health of self, Perceived need for, and effects of, medicine | Not explicitly included in model | Not explicitly included in model | Error producing conditions - poor performance by health professional; Latent conditions -(organisation, systems, culture) | Error-producing conditions - Health of family | Presents human error theory, focuses on individual, organisational and cultural factors; requires further development for understanding of intentional adherence |
| Li, et al. 2005 [50] | To generate culturally sensitive instruments | Cultural health perceptions of Hypertension; beneficial self-care behaviors; Health perceptions of Chinese Herbs; Health perceptions of western medications | Not explicitly included in model | Not explicitly included in model | Not explicitly included in model | Social support | Emphasises “cultural factors” – perceptions, self-care behavior and social support of Chinese immigrants |
| Murray, et al. 2004 [10] | For use in adherence research, and supportive intervention strategies such as pharmaceutical care by pharmacists to improve chronic medication use in older adults | Predisposing Characteristics: age, knowledge, attitudes, beliefs, expectations, perceptual-cognitive resources, health-specific cognitive resources, Need - Perceptions of illness, severity outcome, response to treatment | Medical/ disability-related | Not explicitly included in model | Enabling Resources: Relationship with providers;  Policies, resources, organization, and financial arrangements influencing the accessibility, availability, and acceptability of medical care services | Enabling Resources: Income, distance to health services, transport, insurance, support, supervision; External environment - Patient's home, community composition, level of support derived from these resources | Focuses on patient characteristics, external environment and health system factors; medication-related factors not outlined. |
| Johnson 2002 [51] | To describe the process of medication adherence in individuals undergoing treatment for hypertension | Purposeful action - perceived need, perceived effectiveness, perceived safety; Patterned behavior - access, routine, remembering | Feedback - facts, prompts, events | Not explicitly included in model | Feedback | Patterned behavior - access | The model is a midrange theory which provides specificity about key cognitive (Purposeful Action and Feedback) and non-cognitive (Patterned Behavior) processes of taking medications for chronic illness. |

**Supplementary Table 7: Summary of conceptual frameworks for factors contributing to medication adherence in adults with cancer. Abbreviations: Adjuvant endocrine therapy (AET); Chronic myelogenous leukemia (CML); Healthcare provider (HCP)**

| **Author, Publication Year** | **Model Purpose** | **Patient/ caregiver-related factors in model** | **Condition-related factors in model** | **Medication-related factors in model** | **Healthcare system/ HCP-related factors** | **Socioeconomic factors** | **Comments** |
| --- | --- | --- | --- | --- | --- | --- | --- |
| Adults with **cancer** | | | | | | | |
| Rosa, et al. 2020 [60] | To clarify the concept of analgesic nonadherence for cancer pain and qualify its utility in the context of the opioid crisis | Expectation of pain relief; perceived benefits; self-efficacy; denial of pain as disease symptom; trust in HCP; belief that doctors should focus on cancer treatment rather than pain; socio-demographics; Family hesitancy;  Family characteristics | Older population - Feel better | Type of analgesic; pain relief; type and severity of side effects;  concerns about physiological effects, dependence, addiction | Prescribing practices; Race disparity; complex clinical care, reimbursement, analgesic regulation processes; obtaining analgesics; patient/family burden of coordinating care and assuring effective communication among different providers; insurance, prescription coverage | Not explicitly outlined in model; integrated with patient/caregiver factors and health system/ HCP factors | Categorises antecedents of medication adherence as individual/family level, provider level, and system level; limited attention of condition-related factors in model. |
| Xu, et al. 2019 [61] | To develop a specific belief about health questionnaire for comprehensive evaluation of survivors’ health beliefs about AET | Cognitions and understanding; Self-efficacy; Demographic factors underlying health beliefs - religious beliefs, character, marital status | Recognition of illness recurrence and metastasis | Not explicitly included in model | Behavioral clues for treatment - Timely and effective communication with medical caregivers, Regular information support | Behavioral clues for treatment - Social support from family, friends and other survivors; other socio-cultural factors | Adapted from the health belief model; emphasises health beliefs, demographic factors and socio-cultural factors. Does not account for medication factors. |
| Lambert, et al. 2018 [62] | To describe how personal, social, and structural factors influence AET persistence | Personal beliefs about necessity, recurrence and medications; Balancing quality and quantity of life | Impact on quality of life | Side effects | HCP relationship; Structural factors – support with symptom management, follow-up care | Social support | Presented influencing factors and their inter-relationships; focuses on the balance of quality and quantity of life |
| McGrady, et al. 2016 [64] | To investigate the mechanisms that drive the daily adherence decision-making process among adolescents and young adults with cancer. | Knowledge - purpose, importance; Skills - strategies, prompts/ cues; Decision making - role, preferences; Goals and values | Not explicitly included in model | Side effects, short- and long-term impact, prevention of negative emotions, disruption of normal activities | Medical provider characteristics - trust, communication | Environment & Social network - physical support, encouragement, environment, tools | Presented adherence decision-making as a complex, multi-dimensional process influenced by personal goals and values, knowledge, skills, and environmental and social factors. |
| Verbrugghe, et al. 2016 [63] | To gain insight into adherence behavior in patients taking oral tyrosine kinase inhibitors | Hope, trust, feedback mechanism, anxiety, routine, self-efficacy, knowledge, quality of life, perception of medication properties,  Focus on survival, Focus on quality of life, Balance | Not explicitly included in model | Side effects | Trust based and open relationship with healthcare professionals | social support | Defined three foci (focus on survival, focus on quality of life, and balance between survival and quality of life), influenced by complex, interrelated set of factors |
| McCue, et al. 2014 [65] | Discuss the factors frequently associated with poor adherence | Physical factors; psychological factors; poor literacy; religious/ cultural beliefs; lack of support system; lack of understanding of medication and side effects | Not explicitly included in model | Cost of therapy; Complex treatment regimens; side effects; concomitant medications; Lack immediate treatment benefits | Poor communication with patient; lack of relationship with patient; failure to select appropriate patient for oral therapy; fragmented healthcare system | Not explicitly included in model | Adapted from Jabbour et al. [38] Focuses on barriers to medication adherence; does not consider condition- and socio-economic factors. |
| Gater, et al. 2012 [66] | To inform strategies for improving adherence to oral CML therapies | Patient characteristics; patient knowledge and belief; unintentional factors; behavioral management; perceived benefits of adherence to therapy | High prescription burden, time since diagnosis, temporary other illness, concomitant disease, risk of pregnancy, cancer complexity, complications | Treatment characteristics – dose, duration, side effects, cost, physical properties, drug class; treatment satisfaction; Treatment outcomes | Physician characteristics - Number of active patients in the past year, median duration of first visit with newly diagnosed patient, median duration of follow-up visits, years of professional experience; physician interaction | Lifestyle factors – social situations (alcohol consumption, dining out, travel, holidays) | Presents a complex interplay of factors (including clinical, psychological and behavioral) that influence adherence; limited attention on socio-economic factors |

**Supplementary Table 8: Summary of conceptual frameworks for factors contributing to medication adherence in adults with chronic, non-communicable conditions with asymptomatic and flare phases e.g. rheumatoid arthritis, asthma. Abbreviations: Disease-modifying anti-rheumatic drugs (DMARDs); Healthcare provider (HCP); Inhaled corticosteroids (ICS); World Health Organisation (WHO)**

| **Author, Publication Year** | **Model Purpose** | **Patient/ caregiver-related factors in model** | **Condition-related factors in model** | **Medication-related factors in model** | **Healthcare system/ HCP-related factors** | **Socioeconomic factors** | **Comments** |
| --- | --- | --- | --- | --- | --- | --- | --- |
| Adults with chronic, **non-communicable conditions with asymptomatic and flare phases** e.g. rheumatoid arthritis, asthma | | | | | | | |
| Goh, et al. 2018 [70] | To allow clinicians to remember these factors better and to apply these factors in their daily practice with rheumatic patients | Personality factors; demographics; disease and treatment perceptions; caregiver issues | Mental health; prognosis | Side effects; choice of drugs; signs and symptoms; medical treatment plan | Failure to understand/lack of medical instructions; HCP communication and patient counselling; trust in physician; drug supply | Cost issues; social support | Adapted WHO classification; does not provide information on the weight or magnitude of each factor on adherence rate, |
| Dockerty, et al. 2016 [71] | To better understand why people take or do not take medications for symptom control | Perceived effectiveness of medication; Knowledge and education; perceived use for flares or prophylactic; Perception of osteoarthritis as a disease process vs ageing; perceived patient role in decision making on medication regime | Severity of symptoms - level of pain, frequency, impact on lifestyle, sleeping pattern | Severity and frequency of side effects Acceptability and convenience of prescribed regime | Not explicitly included in model | Not explicitly included in model | Presents a model whereby adherence is perceived as a balance between the willingness and preference to take medications with the alterative being toleration of symptoms; does not account for health system/HCP and socio-economic factors. |
| Voshaar, et al. 2016 [69] | To identify facilitators and barriers of DMARD use in patients with inflammatory arthritis | Capability – knowledge, memory, attention, skills, decision-making process;  Motivation – beliefs about capabilities emotions, motivation and goals, goal conflict | Not explicitly included in model | Environmental context and resources – change of name or appearance of medication, cost of medication | Environmental context and resources - Logistics, access to health professionals, quality of products | Opportunity - Social influences | Captures barriers and facilitators of medication adherence, without exploring the relationships between domains or weight of individual domains. |
| Moshkovska, et al. 2008 [68] | To illustrate the way in which patients appear to balance the benefits and disadvantages of taking 5-aminosalicylic acid (5-ASA) medication | Passive acceptors; Active assessors; Anticipated therapeutic outcome | Seriousness of symptoms | Medication anticipated effectiveness; possible side effects; patients’ experience; Medication regime interference on daily life | Quality of doctor-patient relationship | Not explicitly included in model | Adapted a therapeutic decision model by Dowell and Hudson [29]; Presents the process of assessing medication taken by individuals with ulcerative colitis; limited attention on socio-economic factors |
| Hall, et al. 2007 [67] | To assess patients' perspectives and beliefs about their medication, and its relation to medicine taking and other related health behavior | Fears and concerns; perceived impact of actual or potential symptoms; acceptance of medication and perceived necessity; knowledge and experience; willingness to self-manage | Not explicitly included in model | Not explicitly included in model | Relationship with healthcare provider | Not explicitly included in model | Focuses on medication beliefs; does not account for condition-, medication and socio-economic factors |
| Horne 2006 [72] | For understanding why many patients decide not to use ICS as prescribed | Perceived need; concerns; illness perceptions; background beliefs; contextual issues - past experiences, practical difficulties, self-efficacy, satisfaction | Not explicitly included in model | Not explicitly included in model | Not explicitly included in model | contextual issues - views of others, cultural influences | Employs the necessity/ concerns framework, focuses on influence of perceived necessity/ concerns of treatment |

**Supplementary Table 9: Summary of conceptual frameworks for factors contributing to medication adherence in adults with symptomatic conditions e.g. nocturia and migraine. Abbreviations: Healthcare provider (HCP)**

| **Author, Publication Year** | **Model Purpose** | **Patient/ caregiver-related factors in model** | **Condition-related factors in model** | **Medication-related factors in model** | **Healthcare system/ HCP-related factors** | **Socioeconomic factors** | **Comments** |
| --- | --- | --- | --- | --- | --- | --- | --- |
| Adults with **symptomatic conditions** e.g. nocturia and migraine | | | | | | | |
| Jayadevappa, et al. 2015 [74] | Present a conceptual model to guide the adherence research in nocturia | Demographics; preference, attitude, belief, knowledge | Comorbidity Nocturia bother | Number of medications; Medication type, frequency, duration, efficacy and side effects, follow-up care | Communication, continuity of care, wait time, volume | Geographic and environmental characteristics; income; insurance | A patient-centred model adapted from the Andersen model of health service use; examines predisposition, need and enabling factors. |
| Katić, et al. 2010 [73] | Propose a patient decision-making model to identify migraineurs at high risk for medication adherence problems | Self-efficacy | Importance | Safety | Trust | Not explicitly included in model | Hypothesizes that the best outcomes will occur in the collaborative interactions between a confident patient and a trusted physician; does not account for socio-economic factors |

**Supplementary Table 10: Summary of conceptual frameworks for factors contributing to medication adherence in adults undergoing treatment for chronic, communicable conditions e.g. HIV and tuberculosis in resource-limited countries (Africa, Papua New Guinea). Abbreviations: Antiretroviral therapy (ART); Gay, Bisexual, and other Men who have Sex with Men (gbMSM); Healthcare provider (HCP); Human immunodeficiency virus (HIV); Information-Motivation-Behavioral (IMB); Tuberculosis (TB)**

| **Author, Publication Year** | **Model Purpose** | **Patient/ caregiver-related factors in model** | **Condition-related factors in model** | **Medication-related factors in model** | **Healthcare system/ HCP-related factors** | **Socioeconomic factors** | **Comments** |
| --- | --- | --- | --- | --- | --- | --- | --- |
| Adults undergoing **treatment for chronic, communicable conditions** e.g. HIV, TB in **resource-limited countries** (Africa, Papua New Guinea) | | | | | | | |
| Eshun-Wilson,et al. 2019 [75] | To inform an understanding of ‘why people do what they do’ and assist with future development of patient-centered health services and policies for HIV-positive people in Africa | Level of self-efficacy; acceptance of HIV status | Previous or current HIV-related illness | Conflicting information, messages and views - side effects, scientific uncertainty | Authoritarian health provider; quality of health services | Family and social responsibility;  Poverty, competing priorities and unpredictability; social identity & gender norms; stigma & discrimination; conflicting information, messages and views; support (emotional, logistic, financial) | Supports the ecological perspective of health behavior and represents how engagement is a dynamic process which fluctuates over the long course of HIV care |
| Graham, et al. 2018 [76] | Part of a larger study of an ART adherence support intervention for Kenyan gays, Bisexuals, Men have sex with men living with HIV infection | Intrapersonal – Information, behavioral skills, motivation, resilience | Not explicitly included in model | Not explicitly included in model | Trust in provider;  service provision factors | Interpersonal - support from peers, friends, family, connection to community groups;  Institutional/ Community - gbMSM-friendly services; stigma and discrimination  Sociocultural/Policy – Criminalisation, human rights, funding | Incorporated access-IMB model and socio-ecological model, highlight the marginalized and vulnerable context of this population |
| Diefenbach-Elstob, et al.2017 [82] | To identify factors influencing TB treatment adherence in the remote Balimo region of Papua New Guinea | Confidence in treatment; religious influences; belief in witchcraft;  Use of non-TB standard treatment regimens; Multiple approaches to treatment; Expectations | Presence of symptoms | Side effects/ Lack of effects/ feeling better | Inconsistency in patient education | Strong community network; family and wantok support; expense, duration and difficulty of travel; food availability | Greater emphasis on personal, social and cultural factors |
| Gill, et al. 2017 [77] | Describe attitudes and norms contributing to  adherence for women engaged in care | Behavioral beliefs and attitudes | Prevention of HIV transmission to child; higher CD4 count; | Effects; consequences of non-adherence; lifelong nature | Clinic staff support; | Support from relatives; meeting others living with HIV who exemplified health behavior; others’ negative expressions; stigma/fear of disclosure; social support | Adapted the theory of reasoned action; focuses on behavioral beliefs, attitudes and subjective norms which contribute to intention to adhere and in turn adherence |
| van den Boogaard, et al. 2012 [83] | Explore patient perceptions of adherence to TB treatment | Knowledge, beliefs, reminder cues, intention to adhere, decision to seek biomedical healthcare | Long history of suffering | Not explicitly included in model | Healthcare service factors | Social support, Socioeconomic factors, substance abuse factors, financial/family responsibility | Intention to adhere identified as most important determinant of adherence, preceded by decision to seek health care. |
| Skovdal,et al. 2011 [78] | Develop a framework that can assist with the analysis, planning and execution of ART programmes in other African contexts | Psychosocial dimensions – patient motivation, participation, psychosocial responses to anti-retroviral therapy | Embedded in patient motivation, patient participation psychosocial dimensions | Embedded in patient motivation, patient participation psychosocial dimensions | Embedded in relational contextual dimension | Contextual dimensions - Material, symbolic, relational, institutional support | Identified contextual and psychosocial dimensions influencing adherence |
| Merten, et al. 2010 [79] | Provide synthesis and interpretation of findings of recent social science research on retention in antiretroviral therapy programmes in sub-Saharan Africa | Self-efficacy and identity; Physical, social and mental dimensions of health | Not explicitly included in model | Not explicitly included in model | Medical systems and governance | Negotiating social relationships | Illustrates third order construct of nested relationships of adherence |
| Watt, et al. 2009 [80] | To understand the dynamics of good adherence to ART among patients receiving free ART and HIV-related services from a clinic in Tanzania | Observational learning through role models; Expectation of improved health; Value placed on improved health; motivation to adhere; self-regulation; self-efficacy to adhere despite barriers | Not explicitly included in model | Not explicitly included in model | Reinforcement from healthcare providers | Social support | An explanatory model of ART adherence facilitators, consistent with the constructs of social cognitive theory; does not account for condition- and medication-related factors |
| Nam, et al. 2008 [81] | To identify the psycho-social factors related to adherence behavior in Gaborone, Botswana | Good: Desire to be healthy; Faith; Hope for the future; Ability to make lifestyle changes  Poor: Denial; internalisation of stigma; Belief in traditional healing practices; faith healing; Inability to make lifestyle changes; Fear of being judged by clinic staff Depression; | Good: Recovery from severe HIV illness at start of HIV  Poor: Not sick at time of diagnosis or start of ARTs | Poor: side effects | Not explicitly included in model | Good: Commitment to family as breadwinner; Ability to access social support network; Identifying encouraging confidante  Poor: Inability to access or take food with ARVs; Travel, cost of travel | Adherence pattern is centred on patients’ acceptance/denial of HIV status, rejection/ internalisation of stigma. Little attention on health system/ HCP factors |

**Supplementary Table 11: Summary of conceptual frameworks for factors contributing to medication adherence drawn from empirical data, existing theories and qualitative studies involving adults undergoing treatment for chronic, communicable conditions e.g. HIV, tuberculosis, lymphatic filariasis in countries such as the United States, Europe, Taiwan, Indonesia and India. Abbreviations: Acquired immune deficiency syndrome (AIDS); Antiretroviral therapy (ART); Healthcare provider (HCP); Human immunodeficiency virus (HIV); Highly active antiretroviral therapy (HAART); Information-Motivation-Behavioral (IMB); Lymphatic filariasis (LF)**

| **Author, Publication Year** | **Model Purpose** | **Patient/ caregiver-related factors in model** | **Condition-related factors in model** | **Medication-related factors in model** | **Healthcare system/ HCP-related factors** | **Socioeconomic factors** | **Comments** |
| --- | --- | --- | --- | --- | --- | --- | --- |
| Adults undergoing **treatment for chronic, communicable conditions** e.g. HIV, tuberculosis (drawn from empirical data, existing theories, qualitative studies in United States, Europe, Taiwan, etc.) | | | | | | | |
| Ho, et al. 2020 [84] | To develop a conceptual model for unpacking the complexity of HAART-taking behavior | HIV-positive identity as part of self-identity; values attached to HAART; conscious engagement | Not explicitly included in model | Not explicitly included in model | Not explicitly included in model | Social influence | Focuses on patient factors; did not identify structural factors contributing to medication-taking behavior |
| Engler, et al. 2018 [85] | To produce a conceptual framework for a new patient-reported outcome measure for use in routine HIV care in Canada and France | Cognitive and emotional aspects - affect, beliefs, acceptance, motivation, knowledge; Lifestyle factors - life demands and organisational issues | Health experience and state - body monitoring, manifestations of HIV disease and general health, comorbidity | Side effects, instructions, physical features | HIV clinic and healthcare system issues, pharmacy issues, health insurance;  patient-provider relationship | Social and material context - Social interaction, support and relationships,  HIV stigma and concealment, material and structural challenges; Lifestyle factors - substance use | Illustrates barriers of adherence and their interrelationships |
| Fields, et al. 2017 [86] | To understand adherence barriers among behaviorally infected and perinatally infected youth and develop an intervention specific to their needs | Psychosocial context; intrapersonal level of influence | Not explicitly included in model | Not explicitly included in model | Not explicitly included in model | Psychosocial context | Presents a framework where adherence is affected by multiple levels of influence (as conceptualized in the social ecological framework). Focuses on psychosocial context of adherence barriers and behavior. |
| Dima, et al. 2013 [87] | Exploration of the causes of nonadherence in young Romanian long-term HIV survivors | Information, personal motivation, behavioral skills (self-efficacy) factors are described | Not explicitly included in model | Not explicitly included in model | Not explicitly included in model | Social motivation | Adapted the IMB skills model; modified content to include themes such as role of informational conflicts, long-term goals and altruistic motivation |
| Krentel, et al. 2012 [95] | For identification of key components influencing compliance to mass drug administration for lymphatic filariasis (LF) in Alor, Indonesia | Personal characteristics - health, education, occupation age, sex; Knowledge about LF; personal experience with LF, treatment, health system; Values | Personal experience with LF | Personal experience with treatment and side effects | Personal experience with health system | 1) Individual’s relevant social world  2) Beliefs about society - norms, social reputation, authority/ government, social roles/ gender | Employs causal chain mapping; allows for assessment of factors that take precedence in directing behavior at individual level and across individuals; model is specific to field of mass drug administration for LF elimination. |
| Rongkavilit, et al. 2010 [88] | The proposed modifications to IMB model could be relevant in other cultural settings with more collectivistic worldviews | Information – understanding; knowledge; Behavioral skills - strategies devised; personal motivation | Not explicitly included in model | Not explicitly included in model | Not explicitly included in model | Social motivation, and inter-relational motivation; culture - mutual social support and responsibility, and philosophical Buddhist tenets | Adapted the IMB model with modification to incorporate youths' perceived familial and social responsibilities; does not address condition-, medication-, health system factors. |
| Beusterien, et al. 2008 [89] | To illustrate possible interactions among themes as a tool to help clinicians in their daily management of HIV patients. | Lifestyle fit; emotional impacts | Not explicitly included in model | Regimen complexity/ medication features; side effects; effectiveness | Communication | Communication | Illustrates themes and their inter-dependence; Does not account for condition-related factors. |
| Starks, et al. 2008 [90] | To better understand what is needed to promote optimal ART adherence in China | Proximal cues to action; motivation; knowledge | Not explicitly included in model | Not explicitly included in model | Access – cost of medications, hospital, labs | Access – transportation, time away from work | Based on IMB skills model; four aspects necessary for medication adherence: knowledge, motivation, access, cues to action. |
| Fisher, et al. 2006 [91] | To understand, predict, and promote adherence to HAART regimens | Information; personal and social motivation; behavioral skills; psychological health | Health outcome – viral load, drug resistance, CD4 counts, objective and subjective health status | Not explicitly included in model | Not explicitly included in model | Unstable living conditions, poor access to medical care, service, substance use or addiction, insurance coverage | IMB skills model; illustrates relationships between factors and a feedback loop where health outcomes influence future adherence; does not account for medication- and health system/HCP factors |
| Reynolds, et al. 2003 [92] | provides a framework for understanding how key variables may interact to influence ART adherence behavior | Informational resources; cognitive function and mood state; ART adherence intervention – knowledge, behavioral skills, affective support | Illness experiences (symptoms); HIV health threat; HIV/ AIDS illness/ medication representation, health outcome appraisal | Illness experiences (side effects, regimen complexity) | Interaction with healthcare providers | Interaction with significant others | Adapted from the self-regulation theory; focuses on patients’ illness representation. |
| Wilson, et al. 2002 [93] | To explain how ethnically diverse people with HIV manage interacting symptom clusters and medication side effects as well as their treatment adherence choices. | self-identity, illness ideology; state of mind; adherence choices, personal meaning of time and quality of life | Attributional uncertainty, sometimes silent virus, perceived fickle medical markers | concurrent treatment regimens, medication burden and side effects, impact on lifestyle | Not explicitly included in model | Not explicitly included in model | Adherence is a fluctuating phenomenon; adherence choices are dependent on the state of mind contributed by a particular context and in the face of conditions |
| Barnhoorn, et al. 1992 [94] | Not explicitly indicated | patient characteristics, demographics, pre-disposing factors | Symptoms, health status | Drugs free-of-charge, delivery of drugs at the patient's doorstep | HCP’s attitude about the patient's abilities for change, communication | Income, education, occupation, social and economic aid, support, travel | Conceptualized on the analogy of the original Health Belief Model and DiMatteo and DiNicola’s compliance theory; does not account for health system factors |

**Supplementary Table 12: Summary of conceptual frameworks for factors contributing to medication adherence in adults taking medications for prevention of communicable diseases e.g. prevention of HIV, tuberculosis. Abbreviations: Healthcare provider (HCP); human immunodeficiency virus (HIV); Information-Motivation-Behavioral (IMB); pre-exposure prophylaxis (PrEP); tuberculosis (TB)**

| **Author, Publication Year** | **Model Purpose** | **Patient/ caregiver-related factors in model** | **Condition-related factors in model** | **Medication-related factors in model** | **Healthcare system/ HCP-related factors** | **Socioeconomic factors** | **Comments** |
| --- | --- | --- | --- | --- | --- | --- | --- |
| Adults taking medications for **prevention of communicable disease** e.g. prevention of HIV, TB | | | | | | | |
| Dubov, et al. 2018 [96] | Propose a comprehensive theoretical framework of factors that are likely to influence pre-exposure prophylaxis uptake | Information – objective, subjective; Motivation – risk perception; personal attitude, personal intentions  Behavioral skills – self efficacy, action planning, coping planning; | Psychological ill health | Not explicitly included in model | Not explicitly included in model | Social norms  Moderating factors: lack of insurance coverage, substance use, lack of stable housing, PrEP skepticism (media/ provider), | Adapted the IMB skills model; There are potential relationships between information and motivation constructs, and between motivation and behavioral skills constructs that may need to be further delineated and explored. |
| Jacobson, et al. 2017 [97] | To inform strategies to facilitate successful isoniazid preventive therapy completion among people living with HIV in South Africa | Knowledge, attitude, practices of TB and isoniazid preventive therapy; desire for health preservation; acceptance of HIV diagnosis | Not explicitly included in model | Not explicitly included in model | Healthcare access – trust in healthcare system, resources to access healthcare, efficiency of services | Social support | Focuses on personal, psychosocial and healthcare access factors, and their reciprocal influence |

**Supplementary Table 13: Summary of conceptual frameworks for factors contributing to medication adherence in adults with psychiatric conditions. Abbreviations: Attention deficit hyperactivity disorder (ADHD); Health belief model (HBM); Healthcare provider (HCP)**

| **Author, Publication Year** | **Model Purpose** | **Patient/ caregiver-related factors in model** | **Condition-related factors in model** | **Medication-related factors in model** | **Healthcare system/ HCP-related factors** | **Socioeconomic factors** | **Comments** |
| --- | --- | --- | --- | --- | --- | --- | --- |
| Adults with **psychiatric conditions** | | | | | | | |
| Lim, et al. 2018 [98] | To identify and model medication issues experienced, and coping strategies used by people with dementia and/or family carers | Cognitive and functional ability; knowledge/ education and communication; motivation, attitude, acceptance | Embedded in cognitive and functional ability | Factors relating to obtain and use of medicines, risks of medication error, medication and patient safety; regimen complexity | Not explicitly included in model | Social and cultural factors – lack of appropriate support, caregiver availability, living alone | Modelled medication management issues within four inter‐related system domains - cognitive, medication, social and cultural, and knowledge/educational and communication. |
| Kikkert, et al. 2017 [99] | Useful for clinicians and those involved in the development of adherence interventions or adherence research | Assessment of available options; insight;  self-efficacy; forgetfulness; understanding of the prescription | Embedded in anticipated consequences | Long term effects on well-being (stay well); Short term effects on well-being (feel well); Other indirect effects | Not explicitly included in model | Practical barriers (e.g. finances, accessibility); Support from others | Model shows the dynamic and intertwined relationship between some of the negative consequences of the illness and medication efficacy; requires insight |
| Moitra, et al. 2016 [100] | To illustrate how Acceptance and Commitment Therapy processes might guide the development of more effective adherence interventions for patients with psychotic-spectrum disorders. | Cognitive fusion with stigma and negative thoughts about the illness and treatment, Experiential avoidance of illness-related psychological distress, Values inconsistencies and lack of committed action toward health, Psychological inflexibility in the form of values-inconsistent medication non-adherence | Psychological distress related to the illness and its management, | Complexity, side effect | Not explicitly included in model | Structural barriers – cost, access, transport | Presents a psychological flexibility model of medication adherence that suggests that an individual’s behavior is dependent upon the dynamically changing internal and external environment. |
| O’Callaghan, et al. 2014 [101] | To describe the attitudes, beliefs and experiences of adults diagnosed with ADHD concerning stimulant medication based on the components of the HBM | Self-efficacy; perceived severity (financial problem, job loss, impaired relationship, school failure); beliefs about material, psychological cost, effectiveness of taking medication; Quality of life | Not explicitly included in model | Physical side effects, psychological side effects, lack of effectiveness, increased focus, decreased impulsivity | Cues to action – positive or negative relationship with healthcare professional | Not explicitly included in model | Adapted Health Belief Model; suggested no direct relation between stimulant adherence and quality of life. |
| Gault, et al. 2013 [102] | To explore perceptions of service users and caregivers on adherence and nonadherence to medication in a mental health care context | Refuse medication, loss of credible identity due to hospitalisation denial of disorder; acceptance | Hospitalisation | Not explicitly included in model | ‘Play the game’ to escape from hospital; professionals’ response – use of collaborative decision making | Not explicitly included in model | Emphasises that the behavior of professionals is crucial in collaborative decision making on medication adherence; does not account for medication-related factors. |
| Sanders, et al. 2013 [103] | To promote adherence to methadone maintenance treatment | Lack of control/ participation in treatment, disdain for getting high, concern about establishing new drug habit | Medical conditions | Intrinsic – notion of drug doses being too high, desire to avoid side effects, need to avoid withdrawal symptoms or dope sickness, methadone form; medicine interaction | Not explicitly included in model | Family and peers, shame and stigma | Model of comfort, function, perceived dose appropriateness; considers intrinsic and extrinsic factors exerting upward and downward pressure on perceived dose appropriateness and adds nuance to understanding of the acceptability of certain doses |
| Hon 2012 [104] | To gain a greater understanding of patients’ medication-taking practices, and their subjective experiences with antipsychotic treatment | Quality of life; health status; Discernment | Health status – Impact of illness, | Health status – effects of medication | Not explicitly included in model | Not explicitly included in model | Emphasised quality of life as the core category, with centralising influence on medication taking behavior, and has relations with health status and discernment. |
| Roe, et al. 2009 [105] | To explore why and how people with a serious mental illness choose to stop taking prescribed medication | Internal experience; doubts, resources and confidence regarding stopping medication; personal perspective on use of medication, feelings evoked | Experience major emotional crisis | Subjective experience of taking medication | Doctor-patient relationship | Not explicitly included in model | Emphasize the importance of the doctor-patient relationship context; socio-economic factors not included in model |
| McCann, et al. 2008 [106] | Present a self-efficacy model of medication adherence in chronic mental illness | Personal issues; Self efficacy Perceived medication efficacy | Not explicitly included in model | Medication side effects and complexity; | Access to and relationships with health professionals | Social stigma;  Supporting living circumstances; Significant other support | Gives prominence to self-efficacy, medication efficacy and immediate social, psychological and environmental supports; limited attention on condition-related factors. |
| Corrigan 2002 [107] | For development of a strategic set of interventions that will improve adherence to anti- psychotic medications | Value expectancies | Lack of disease awareness; cognitive deficits; reactance to disempowerment | Medication side effects | Poor alliance; insufficient adherence information | Lack of resources/ support to obtain/ maintain medications; family/ friends who do not support medication: | Based on health belief theories; emphasises disabilities of psychoses, value expectancies and social support in explaining medication adherence. |
| Perkins 1999 [108] | Using the health belief model, clinicians can assess the relative impact of various factors on medication adherence | Risks of illness: insight, perceived severity of symptoms, cues to act | Disease related barriers: cognitive impairment/ disorganisation, paranoid delusions/ suspiciousness, substance abuse | Complex regimen; Benefits and costs of treatment are described | Healthcare system inadequacies | Lack of transportation, financial difficulties | Modified Health Belief model; posits that adherence to treatment is determined by the patient’s assessment of the perceived benefits of treatment and risks of illness versus the costs of treatment |
| Fenton, et al. 1997 [109] | Elements of this modified health belief model outline  areas of assessment pertinent to the differential diagnosis of noncompliance | Goals and priorities; cues to action; cognitive and motivational resources to assess risk and formulate action; perceived adverse effects of illness and personal risk of suffering effects; Perception of effectiveness and feasibility | Psychopathology | Side effects | Not explicitly included in model | Substance abuse; Finances;  Transportation | Modified Health Belief model for schizophrenia, incorporated cognitive and motivational resources to assess risk and formulate action |
| Davidhizar 1984 [110] | Examines the interface of the reinforcement/ punishment model and adherence and nonadherence by clients with schizophrenia to medication regimens | Opportunity to do desired things; personal freedom, autonomy; avoidance of hospitalisation, commitment, seclusion and restraint, force to take medication | Improvement in cognitive thinking ability; symptoms; avoidance of psychoses | Side effects; dysphoric effects of drugs; drug induced “normalcy” | Reaction from doctor, staff | Reaction from family, friends | Employs operant conditioning model of reinforcement and punishment to explain adherence; does not readily provide an explanation of all aspects affecting adherence. |
| Jamison, et al. 1983 [111] | Develop a clinical framework that considers the complex interaction of variables in determining noncompliance | Idea of having lifetime disorder and necessity of daily mediation; perceived cost-benefit varies with gender; | Mood swings; impaired creativity | Side effects | Ambivalence; attitude towards bipolar illness, patient education | Cultural factors – anti-drug attitude; misrepresentation in media | Discusses interaction of patient, illness, medication and socio-cultural factors on adherence; does not account for health system factors. |

**Supplementary Table 14: Summary of conceptual frameworks for factors contributing to medication adherence in pediatrics patients. Abbreviations: Healthcare provider (HCP); Attention deficit hyperactivity disorder (ADHD); Antiretroviral therapy (ART); Combination antiretroviral therapy (cART); Capability, Opportunity, Motivation-Behavior (COM-B); Human immunodeficiency virus (HIV); World Health Organization (WHO)**

| **Author, Publication Year** | **Model Purpose** | **Patient/ caregiver-related factors in model** | **Condition-related factors in model** | **Medication-related factors in model** | **Healthcare system/ HCP-related factors** | **Socioeconomic factors** | **Comments** |
| --- | --- | --- | --- | --- | --- | --- | --- |
| **Pediatrics patients** | | | | | | | |
| Heneghan, et al. 2020 [119] | To use the COM-B to model oral chemotherapy adherence in pediatric acute lymphocytic leukemia | Capability – physical; psychological; motivation - automatic and reflective | Not explicitly included in model | Not explicitly included in model | Opportunity –physical (e.g. contact child’s doctor, access health records) | Opportunity – social; physical (e.g. pay for medications and all appointments) | Employs Capability, Opportunity, Motivation-Behavior (COM-B) model; limited attention to condition- and medication related factors |
| Giralt, et al. 2019 [112] | Assess multi-level factors influencing caregivers’ acceptance of and adherence to lopinavir/ritonavir pellets as well as their underlying mechanisms | Information, adherence support; administration routines; self-efficacy; stress reduction; coping; acceptance for all caregivers; trust; Autonomy; | Not explicitly included in model | Better taste; dosing method; possibility to mix pellets with water or tea; easy storage; experience of positive effects | Not explicitly included in model | Informal social support; Reduced indirect cost; reduced risk of stigmatisation | Examines interaction between factors; does not account for condition- and health system/HCP factors |
| Khan, et al. 2019 [120] | To identify the factors affecting the three phases of medication adherence in people  with ADHD | Family, caregiver and patient characteristics, beliefs are documented | Number, severity of symptoms; time since diagnosis; Functional remission | Frequency, duration, cost, effects, dosage, count, type, administration | Health professional's advice; Relationship with physicians, medical facility | Conflicting information, media portrayal, socio-economic status, social stigma, community support, geographic variation | Examined factors influencing three phases of medication adherence |
| Galea, et al. 2018 [113] | Investigate the barriers and facilitators to cART adherence among Peruvian adolescents living with HIV | Life stage issues; emotional state; lack of/ misinformation; personal strategies; plans for the future; adult support  suboptimal relationship with caregivers; rewarding cART adherence; Provision of information on HIV/cART | History of declining health due to suboptimal cART adherence | Negative experiences with cART | Health system; hospitalisation for intensive support; peer support | caregiver's economic resources; | Employs socio-ecological model; medication factors conceptualised from perspective of patient behavior, may underemphasize the need for pediatric-friendly cART options |
| Goh, et al. 2017 [118] | A baby bear model was proposed to  better visualize five categories that affect cancer treatment adherence | Patient’s Personality Factors; Demographics; Disease and Treatment Perceptions; Caregiver Issues | Concrete thinking; Poor prognosis | Side effects; Length and complexity; Route of administration and properties of tablet | Understanding medical instruction; access to health insurance; supportive presence;  poor HCP communication;  perception of hospital care; inadequate drug supply | Financial difficulties Transportation issues Presence of more siblings | Adapted from WHO classification; inconclusive of whether financial difficulty is most strongly associated with non-adherence in pediatric oncology population |
| Sonney, et al. 2016 [117] | Allow researchers to better understand parent-child shared management of asthma | Parent and child illness representation; coping procedures, action plans, appraisal | Health threat; illness representation – identity, timeline, consequences | Embedded in illness representation – Effectiveness of treatment | Not explicitly included in model | Not explicitly included in model | Modification of the Common Sense model of Self-regulation to incorporate illness representation of parent and child; does not account for health system and socio-economic factors |
| Olds, et al. 2015 [114] | To understand the caregiver’s and child’s experiences with anti-retroviral adherence, barriers and facilitators of adherence, role of social support for the caregiver | Child-caregiver relationship; Child’s personal responsibility; Caregiver's sense of obligation and commitment | Lazarus effect – a major event that impressed upon caregivers the importance of anti-retroviral medications and the necessity of adherence | Embedded in the Lazarus effect | Not explicitly included in model | Lack of resources; private and institutional structural social support; Emotional and informational support | ‘Lazarus effect’ as the initial motivation for adherence; caregiver’s obligation/ commitment promotes adherence over time. Does not account for health system/ HCP factors. |
| Haberer, et al. 2009 [115] | To improve the understanding of the complex challenges and to identify potential areas for intervention with this vulnerable population urgently in need of support | Child characteristics; Caregiver & family characteristics; | Not explicitly included in model | Regimen characteristics | Not explicitly included in model | Social and cultural characteristics | Presents four major influences of pediatric medication adherence –child, caregiver and family, regimen, social and cultural characteristics |
| Vreeman, et al. 2009 [116] | To explain how childcare for HIV-infected children on anti-retroviral therapy (ART) in western Kenya is sustained | Child – health status, refusing medicines, developmental state, position in family; parent/ caregiver – relationship to child, health status, coping mechanism; Information sharing; ethnicity | Not explicitly included in model | Not explicitly included in model | Healthcare system - Counselling skills  treatment decision making, attitude toward patients  personal beliefs about HIV, approachability, access to clinics and medicines | Household – support and resources; community – beliefs, disclosure, stigma, isolating, encouraging/ helping; Information sharing and social interaction | Employs ecological model of pediatric ART adherence, with processes of information-sharing and social interaction that integrate domains or lead to disintegration of the connections |
